# Silicon Era of Carbon-Based Life: Application of Genomics and Bioinformatics in Crop Stress Research

**DOI:** 10.3390/ijms140611444

**Published:** 2013-05-29

**Authors:** Man-Wah Li, Xinpeng Qi, Meng Ni, Hon-Ming Lam

**Affiliations:** Center for Soybean Research, State Key Laboratory of Agrobiotechnology and School of Life Sciences, the Chinese University of Hong Kong, Shatin, N.T., Hong Kong; E-Mails: limanwah@cuhk.edu.hk (M.-W.L.); qixinpeng@cuhk.edu.hk (X.Q.); nimeng@cuhk.edu.hk (M.N.)

**Keywords:** bioinformatics, crops, genomics, stresses

## Abstract

Abiotic and biotic stresses lead to massive reprogramming of different life processes and are the major limiting factors hampering crop productivity. Omics-based research platforms allow for a holistic and comprehensive survey on crop stress responses and hence may bring forth better crop improvement strategies. Since high-throughput approaches generate considerable amounts of data, bioinformatics tools will play an essential role in storing, retrieving, sharing, processing, and analyzing them. Genomic and functional genomic studies in crops still lag far behind similar studies in humans and other animals. In this review, we summarize some useful genomics and bioinformatics resources available to crop scientists. In addition, we also discuss the major challenges and advancements in the “-omics” studies, with an emphasis on their possible impacts on crop stress research and crop improvement.

## 1. Introduction

According to the Food and Agricultural Organization of the United Nations (FAO), food production must be increased by 70% in the next 40 years to meet the increasing global demand [1]. Abiotic and biotic stresses are major limiting factors hampering crop productivity. Therefore, understanding the stress responses of crops using genomic information is important in bringing forth more effective crop improvement strategies.

**Figure 1:**
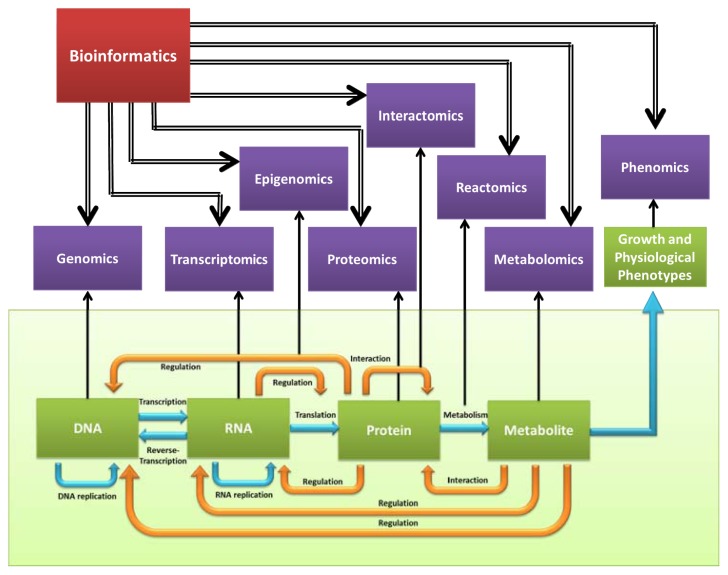
Infusion of biological “-omics” with bioinformatics.

The publishing of the *Arabidopsis thaliana* genome in 2000 is a cornerstone of the plant genomics era [2]. Taking advantage of the high-throughput data acquisition platforms of the next generation sequencing technology, additional crop genomes have been subsequently decoded. So far, the draft genomes of more than 40 plants have been completed, including those processed in the 1000 Plant and Animal Project [3]. Other “-omics” technologies such as transcriptomics, proteomics, metabolomics, and phenomics (Figure 1) have also undergone rapid development in recent years. Together, there is a large volume of accumulated data, and hence data management and data mining have become a bottleneck for “-omics” researches.

To convert the great amount of data into manageable information, it is essential to establish standard formats and methods for storing, retrieving, and sharing data. Algorithms based on mathematical and statistical models are needed to handle biological data. This review aims to provide a systematic summary of the currently available databases and bioinformatics resources and highlight some challenges and advancements in the study of genomics and other “-omics”, with emphasis on their implications on crop stress research.

## 2. General Bioinformatics Resources

### 2.1. Databases

Various databases have been developed to accommodate the comprehensive -omics data and some of them also provide onsite analytical tools (Table 1). The three commonly used sequence databases are GenBank in USA, European Nucleotide Archive (ENA) in Europe, and DNA Data Bank of Japan (DDBJ). They are collaboratively accommodated by the International Nucleotide Sequence Databases (INSD), and the deposited data are frequently synchronized. There are also repositories designated specifically for plants, such as Phytozome that holds the genomic information of more than 40 plant species, including all the sequenced crops. Besides basic genomic information, databases such as Legume Information System (LIS) facilitate synteny analyses and comparative genomic studies between closely related crop plants.

Online resources for individual crops, together with massive datasets, have been developed (Table 2) where systematically integrated information including: genetic resources (genetic maps, molecular markers, and quantitative trait loci (QTL)); genomic resources (DNA sequences, gene models, and regulatory elements); gene expression data (ESTs, cDNA sequences, and transcriptomes); and functional units (proteomic and metabolomic data), is provided. Crops of higher economic values are usually accompanied with a more comprehensive database. The genomic sequences of some economically less important crops, such as foxtail millet, sorghum, and barley, have been released recently [18–20] and their corresponding integrated databases are still under development.

Some data repositories also provide information related to abiotic and biotic stress responses. For example, in MaizeGDB, there are well documented records for tropical maize exhibiting tolerance to drought stress [21]. In SoyBase, genetic markers associated with salt tolerance, drought tolerance, and cyst nematode resistance are incorporated with genomic and expression information. Databases for individual crops could also facilitate the unveiling of the genetic basis of specific traits. For example, the tomato genome sequence helped identify the R-genes which were then incorporated in the Plant Resistance Genes database [22].

### 2.2. Biological Ontologies Related to Crop Stress Research

The standardization of ontology is important for the structuring of huge datasets, interconnection between databases, merging resources, and curation of information. Each ontology term has its own name, identifier/ID/accession number and definition. The identifier/ID/accession number is usually made up of a prefix and a number. For example, the Gene Ontology term “lipid binding” has the accession number GO:0008289. The definition of “lipid binding” is a gene product that can interact selectively and non-covalently with a lipid.

The Gene Ontology (GO) project provides a well-established and controlled vocabulary database for describing the function of a gene and its gene product. The ontology covers three aspects, including cellular component, molecular function, and biological process. GO is used in genome annotation to provide information on gene products. An evidence code (by Evidence Code ontology) is used to describe the evidence that links the GO annotation with the gene product. The Evidence Ontology (EO) suggests whether an annotation has been made manually by a curator or by automated electronic annotation. For example, EXP refers to: “inferred from experiment”; IBA refers to: “inferred from biological aspect of ancestor”; and IEA refers to: “inferred from electronic annotation”. All this information can be found in the Gene Ontology website [27].

Plant Trait Ontology (TO) is a controlled vocabulary for describing the plant trait and phenotype. In addition to anatomical and morphological traits, TO also includes a subset of controlled vocabularies for abiotic and biotic stress traits. For example, the yellow dwarf disease resistance (TO:0000292) is the child term of resistance to disease by mycophasma-like organism (TO:0000013) under the lineage of stress trait (TO:0000164).

There are many other biological ontology projects for different research fields. Ontologies listed in Table 3 contain the information related to crop stress responses.

## 3. Recent Advances and Challenges in Crop Genomics

### 3.1. Polyploidy as a Major Challenge in Crop Genome Assembly

Polyploidy is a major hindrance in crop genome assembly. One of the ways to tackle the highly polyploid genomes is to make references to the closely related, putative progenitor diploid genomes if they are available. The Catalogue of Life [32] and the Integrated Taxonomic Information System [33] may help to identify such related species. For example, the fiber-producing cotton (*Gossypium hirsutum*) is tetraploid, comprising an A-genome and a D-genome. To assist the assembly of the tetraploid genome, the diploid D-genome of *G. raimondii* was first sequenced and assembled [34]. A second example is strawberry (*Fragaria × ananassa*), with an estimated genome size of about 600 Mb. Although this is much smaller than other crop genomes, it is an octaploid (AAA′A′BBB′B′) [35]. Therefore, the genome sequence of the woodland strawberry (*Fragaria vesca*), a potential progenitor of *Fragaria × ananassa*, was completed in 2012 to provide the first diploid model for the genomes of *F.* spp. [35,36]. Wheat is another example of polyploid crop genomes. The hexaploid bread wheat (*Triticum aestivum*) contains the A, B and D genomes, which probably originated from *Triticum urartu* (A genome), *Aegilops tauschii* (D genome), and an unknown species related to *Aegilops speltoides* (B genome). The genomic sequence information of *T. aestivum*, *T. monococcum* (a community standard line related to the A-genome donor), and *Ae. Tauschii*, as well as the cDNA sequence information of *T. aestivum* and *Ae. Speltodies*, were obtained [37]. With reference to the respective diploid genome information, over 90% of the wheat genes were successfully assembled into the A, B, or D genome with over 70% precision [37]. The drafted *de novo* genomes of *T. urartu* and *Ae. tauschii* were recently published, representing 94.3% and 97.0% of the predicted genome sizes respectively [38,39]. Although the lack of a good reference for the B genome is still an obstacle in building the *T. aestivum* genome, these pieces of work have built a good framework for the further whole genome assembly of bread wheat, and established a model for the study of other polyploid genomes.

### 3.2. Reduced Genetic Diversity of Modern Crops

Modern crops originated from a small number of plants. Bottleneck effects during domestication and prolonged human selection together have significantly reduced the genetic diversity of modern crops. Such a reduction in genetic diversity has been confirmed by several genomic studies (Supplementary Table S1). For example, whole-genome resequencing of 14 cultivated and 17 wild soybean genomes revealed that the wild soybeans have higher numbers of SNPs and genetic diversity compared to those of the cultivated ones [40]. The domesticated rice cultivars (*Oryza sativa* indica and *Oryza sativa* japonica) also show a lower genetic diversity than their wild relatives (*O. rufipogon* and *O. nivara*) in a study on 50 accessions of cultivated and wild rice [41]. More interestingly, even though both indica rice and japonica rice are cultivated, the japonica rice shows significantly lower genetic diversity than the indica rice, suggesting that the japonica rice has suffered from a stronger bottleneck effect under domestication [41]. On the other hand, although maize landraces and improved lines have retained a higher nucleotide diversity from their wild progenitor, as compared to other self-fertilizing crop species, a weak bottleneck effect can still be observed [42]. Reduced genomic diversity of major staple crops limits their adaptability to the changing environment and reduces the room for crop improvement. Therefore, crop improvement programs should turn their focus to the genetically compatible wild species, which have higher biodiversity and can serve as natural genetic reservoirs.

### 3.3. Sequence and Structural Variations in Genomes Providing Clues for Stress Studies

Sequence differences and structural variations in genomes are usually identified by comparing the genomes of wild species to their related landraces and modern cultivars, and also to other model plants. These differences can, on the one hand, provide information about genome evolution, and, on the other hand, serve as molecular markers for genetic mapping. Sequence differences and structural variations that affect gene structure, gene expression, and gene copy number are major determinants shaping the diversity among different varieties of the same species. For instance, wild soybeans and some rice accessions possess some present/absent variations or unmapped contigs that contain *bona fide* genes annotated to be involved in abiotic and biotic stresses [40,41,43,44]. One specific example relating to biotic stresses is the enrichment and over-representation of LRR (leucine-rich repeat) and NB-ARC (nucleotide-binding adaptor shared by APAF-1, certain *R* gene products and CED-4) domain-containing genes in some crop genomes [19,45]. In plant genomes, disease resistance (*R*) genes are responsible for defense responses [46]. LRR and NB-ARC are two important domains found on the R proteins [46]. The LRR domain-containing proteins play important roles in pathogen-host interactions and the activation of defense responses [47,48]. On the other hand, the NB-ARC domain is responsible for the mulitmerization and autoactivation of the R proteins upon stimulus [49]. The LRR and NB-ARC-containing genes exhibit higher ratios of nonsynonymous-to-synonymous SNPs than the genome average in crops such as soybean [40], rice [41,50], and sorghum [51]. In maize, 101 out of 3490 large-effect SNPs detected are located on 49 LRR domain-containing genes [44]. LRR and NB-ARC domain-containing genes are important components in the plant defense response system [46,49,52] while the high nonsynonymous-to-synonymous SNP ratio of LRR or NB-ARC domain-containing genes suggests a dynamic evolution of these genes to combat pathogens.

In addition to disease resistance genes, some transcription factors are found to be over-retained after the whole-genome duplication in *Musa* α/β (banana) [53]. Some of these transcription factors such as Myb, AP2/ERF, and WRKY are known to be important regulators in abiotic stress responses [54]. On the other hand, compared to rice, sorghum, and maize, there are more genes encoding for cytochrome P450, CCAT-binding factor transcription factors, late-embryogenesis-abundant proteins, and osmoprotectant biosynthesis proteins in the *Ae. tauschii* genome (progenitor B genome of wheat) [38]. These genes are important for the adaptation to cold and physiological drought. Moreover, a significantly higher number of transmembrane ATPase subunits, which are probably involved in Na^+^ exclusion and mineral uptake, have been detected in *Ae. tauschii* than in wheat [37,38]. The extra genes in *Ae. tauschii* may be good candidates for wheat improvement.

### 3.4. Advances in Ultra-High-Density Genetic Mapping Using SNPs

Genetic mapping using genetic populations is one classical strategy to identify genes related to stress responses. Members in the mapping population can either be related (e.g., QTL mapping using bi-parental populations) or unrelated (e.g., genome-wide association study (GWAS) using germplasm collections) (for population structure, data characteristics and methods, see reviews [55,56]). There are some successful cases in identifying stress tolerance causal genes through mapping [57–59]. For example, a salt tolerance-conferring sodium transporter from rice was identified through QTL mapping [58]. The *SKC1* locus corresponding to shoot K^+^ content was mapped with a BC_2_F_2_ population generated from a cross between a salt-tolerant indica variety and a susceptible japonica variety [58]. The *SKC1* locus was further confined to a 7.4-kb stretch by the BC_3_F_4_ progeny testing of fixed recombinant plants. The locus contains only a single open reading frame, which encodes for a HKT-type transporter. *SKC1* near-isogenic lines accumulated less Na^+^ under salt treatment compared to the susceptible parent. Voltage-clamp also supports the notion that the SKC1 protein functions as a Na^+^-selective transporter that probably regulates K^+^/Na^+^ homeostasis under salt stress [58].

Classical molecular markers for mapping such as AFLP, RFLP, and SSR markers are sparsely distributed in the genome, and hence limit the mapping resolution and pose difficulties in pinpointing the phenotype-causal genes. With the availability of genomic sequence data, SNP markers become more accessible for use in mapping, to help achieve a much better resolution. However, conventional PCR-based methods are laborious and time-consuming while the resolution of array-based methods is limited by the number of probes on the array.

High-resolution genotyping by whole-genome resequencing has been established [60,61], making the ultra-high-density genetic mapping more attainable. In principle, this method can achieve the highest resolution, provided that there are enough resources to capture all the SNPs in a population. In reality, polymorphic SNPs are usually captured by low-coverage sequencing (~1X for unrelated populations [58] and <0.1X for recombinant populations [60,62,63]).

In a QTL study of recombinant inbred populations originating from indica and japonica rice, SNPs between the parental reference genomes were first identified using DiffSeq in the EMBOSS package and cleaned by SSAHASNP in the ssaha2 package. Low-depth sequencing reads of recombinant inbred lines (RILs) were mapped to the parents’ pseudomolecules by using the SSAHA2 software [64] to determine the genotype of each RIL. SNPs were analyzed by a sliding window approach to determine the recombinant break points within the genome of every single line in the population to form a bin map [60]. This sliding window strategy can accommodate the high error rate of next generation sequencing and allow missing data resulting from low-coverage sequencing [60]. Each “bin” will serve as a “marker” in the subsequent linkage map construction using MAPMAKER/EXP and in QTL mapping using QTL Cartographer. In this study, using 150 rice RILs, the sequencing-based method increased the resolution by 35-fold and greatly reduced the time needed for genotyping, compared to the map generated from 287 PCR-based markers [57]. The power of this method was further illustrated in a study using 210 rice RILs to map the *GS3* and *GW5*/*qSW5* loci related to the grain length and grain width, respectively [62].

Since missing genotypes in low-depth sequencing would reduce the effectiveness of GWAS, after SNPs have been identified by mapping the sequencing reads, the *k*-nearest neighbor method (KNN) that uses in-house algorithms for data-imputation can be adopted in addition to increasing the sequencing depth, in order to reduce the missing genotypes [61]. GWAS has been conducted in mapping 14 agronomic traits, including drought tolerance, using 373 indica rice lines. One to seven loci have been mapped for each trait, and some of them overlap with the previously known loci/genes identified through bi-parental QTL mapping or mutant studies [61]. With the great reduction of sequencing cost (<US$0.1 per raw megabase in 2012) [65], we anticipate that mapping by sequencing will become a popular method to obtain high resolution maps for stress-related loci/genes.

### 3.5. Genomic Selections

Genomic selection (GS) is introduced to evaluate the overall effects of all contributing loci genome-wide [66]. During the process of GS, a training population will be used for computational model training to obtain the genomic estimated breeding values (GEBVs) [67]. Complex traits such as drought tolerance are usually determined by multiple small-effect QTLs. GEBV associates markers and QTLs by regarding all the markers as variables contributing to the trait and the effect of each marker allele towards the complex QTLs is quantified (it can be zero). GEBV determines the sum of the marker effects and thus indicates the breeding value of an individual; favorable individuals with high GEBVs from breeding populations will be selected for field application. Genotypic and phenotypic information of the breeding population can be used to further improve the computational model to form a training-breeding cycle [66]. Unlike GWAS and QTL studies, which are designed to reduce the breeding time by selecting plants with desired molecular markers at early growth stages instead of evaluating the actual phenotypes at a later stage, GEBVs serve only as selection criteria but do not lead to target markers or causal genes.

As high-throughput genotyping and phenotyping have accelerated GS studies by increasing marker density and selection capacity, one of the major challenges of GS is selection accuracy. Evaluations of GS accuracy have been performed in maize [68], wheat [69,70], barley [71], and cassava [72]. Several statistical models for GEBV calculations, including best linear unbiased prediction (BLUP) [67], Bayesian shrinkage regression (BayesA, BayesB, *etc.*) [73], and mixed models have been employed. There is no agreement on which model is the most efficient, because many factors such as population size and genetic background may affect statistical power [71]. It is believed that GS is a valuable approach for plant breeding [74], however, it will take some time for this concept to develop into a practical tool [75]. A GS-based breeding scheme has already been proposed and is considered to be an important tool for developing durable stem rust-resistant wheat [76].

### 3.6. Identification of Stress-Related Gene Families

When properly annotated genomes are available, the genome-wide identification of all members of a gene family will become feasible. Since genome duplication (polyploidy or paleopolyploidy) and single gene duplication are common in crops [77], genes usually exist in multiple copies and/or in gene families. Identifying all members of a gene family may give a more comprehensive view on the possible functions of a group of evolutionarily related genes. Bioinformatics tools such as Fgenesh [78], GAZE [79], and JIGSAW [80] have been adopted for searching gene families in crops.

Two typical ways to identify members of gene families from within a genome are keyword search and pattern/homology search. Keyword search usually requires precise keywords including gene names and controlled vocabularies. The most commonly used controlled vocabularies are Gene Ontology, as mentioned in section 2.2, and the functional classification by Pfam, InterPro and KEGG [81–83].

A genome-wide pattern search usually begins with searching sequence databases using programs like BLASTP or TBLASTN [84]. Databases can either be online resources (Table 1) or in-house databases. The occurrence of the desired functional domains in the potential sequences can then be verified using the Pfam protein families database [81], SMART database [85], or HMMER [86]. When the BLAST results are associated with unannotated sequences, these will require further analyses to determine the putative gene structures. One example of applying the above strategy to identify stress-related genes is the analysis of AP2/EREBPs in the rice genome [87]. “AP2/EREBP” was used as the keyword in searching databases, including DRTF, MSU NCBI, and KOMBE. Any non-redundant sequences obtained were then used as query terms in the TBLAST and BLASTP searches of the MSU and NCBI databases. Four genes with an incomplete AP2 domain were excluded after Pfam and SMART analyses because of their very small AP2/ERF domain. A total of 163 genes were identified using this method, in contrast to the 139 genes as suggested previously [87]. Expression studies revealed that a number of the members are responsive to abiotic or biotic stresses. A few of them can even be induced by multiple stresses, suggesting their possible involvement in stress responses [87].

Supplementary Table S2 summarizes the strategies and tools used in recent literature on genome-wide analyses of gene families related to stress responses in major crops.

## 4. Functional Genomics

### 4.1. Transcriptome

There are two major technologies for obtaining the overall transcription map of specific plant tissues: hybridization-based microarray technology [88] and next generation RNA sequencing technology (RNA-seq) [89]. RNA-seq technology, in conjunction with efficient bioinformatics tools, is now more widely used to support predicted gene models, extract differentially expressed genes, and find novel transcripts in *de novo* assemblies. Public repositories such as ArrayExpress [90] are designed for the storage of expression data. Standard data formats including Minimum Information about Microarray Experiments (MIAME) or Minimum Information about Sequencing Experiments (MINSEQE) are unified to facilitate transcriptome data submission/downloading. Bioinformatics tools dealing with transcriptome alignment, splicing event prediction, and *de novo* assembly are also available (Table 4).

Crops such as maize [100] and soybean [101] have their own transcriptome atlases, compiled from sub-transcriptomes from multiple tissues and different developmental stages. For the transcriptome atlas of soybean, plant ontology (PO) was used to describe the developmental stage of each experimental tissue, providing a common ground for readers and users to discuss and perform further analyses. The cDNA short reads generated by Illumina Genome Analyzer were aligned to the soybean reference genome sequence assembly using GSNAP, released in 2005. The digital expression counts were determined using the R programming language and normalized using a variation of RPKM methods [101]. The global inventory of expressed transcripts of crops under stress is dynamic, both temporally and spatially. Time series sampling is a typical experimental design to trace the trajectory of such differentially expressed transcripts of crops under stress conditions. A typical example was the study of the soybean transcriptome under alkaline stress. Soybean plants were treated with NaHCO_3_ and transcriptomes were analyzed using microarray [102]. GO terms were successfully assigned to the 1380 significantly changed probe sets that are related to metabolism, signal transduction, energy, transcription, secondary metabolism, transporter, as well as disease and defense. A time series study revealed the interplay of signal transduction and metabolism during the progression of the treatment. MapMan tools were used to visualize these changes [102]. Other time series studies include the studies of rice root under low potassium [103], cassava under cold stress [104], and soybean subjected to *Pseudomonas syringae* infection [105]. The other widely reported experimental design is the comparative transcriptome study performed among crop accessions with different degrees of stress tolerance, such as the study of soybean accessions exhibiting differential tolerance toward low potassium [106], rice cultivars with contrasting abilities to withstand drought [107] and chilling [108], wheat with differential drought tolerance [109], and Medicago [110] and foxtail millet [111] cultivars with differential salt tolerance.

Another strategy to associate transcript abundance to genomic variations is the expression QTL (eQTL), which use differentially expressed transcripts as the quantitative traits [112]. The eQTL maps of maize root [113] and rice shoots [114] have identified thousands of *cis* and *trans* regulation factors by population transcriptome screening. The eQTLs co-localizing with traditional QTL regions could give supportive evidence explaining the genetic basis of the targeted phenotypic characters. One successful example is the eQTL study of the partial resistance toward *Puccinia hordei* in barley [115], in which some eQTLs were reported to co-localize with previously known rust resistance QTL regions.

### 4.2. Proteome

Due to the alternative splicing of RNA transcripts and post-translational modifications of the proteins themselves, the proteome within a cell can be much more complicated than the corresponding genome. The gel-based proteomics technology will soon be obsolete due to its limited sensitivity and semi-quantitative nature [116]. The rise of the next generation proteomics systems such as Orbitrap and QStar, together with the application of isotopic tag-based quantitative proteomics (ICATs [117], SILAC [118], isobaric tag-based quantitative proteomics (ITRAQ [119]), and label-free quantitative proteomics (MaxQuant [120], Serac [121], SIEVE (Thermo Scientific, San Jose CA, USA)) have expedited the development of high-throughput proteomic studies. Nevertheless, the pace of adopting these platforms in plant stress studies is far behind studies in humans.

Despite the advancement in the proteomics platforms, the application of *de novo* peptide sequencing is still limited. Protein identifications still largely rely on database searches in which experimental peptide mass spectra are compared with theoretical peptide mass spectra generated from existing sequence databases. Some commonly used databases and useful algorithms are summarized in Table 5. Since the genomes of many crops have not been completely sequenced, and some others are still unknown, proteins of species without a genome database are frequently identified by referring to cross-species databases. In these cases, it is not uncommon that molecular weights and isoelectric points (pI) of the identified proteins may deviate from the actual spot position on the 2D gel, despite the high protein scores.

Comprehensive reviews summarizing plant proteomic studies from 2006 to 2008 are available [122,123]. We have also summarized the plant proteomic studies in 2012 (Supplementary Table S3). Recently, plant proteomic investigations have been subdivided into several areas, including subcellular proteomics and proteomics-related post-translational modifications. For example, 21 differentially expressed proteins were identified from salt-treated wheat chloroplasts [124], and 13 and 11 differentially expressed microsomal proteins, respectively, were identified from two distinct cadmium-accumulating soybean cultivars [125].

Stress-induced posttranslational modifications of proteins are common. They are either the results of deleterious damage from the stress, or beneficial modifications to regulate the functions of the proteins in order to cope with the stress. To study posttranslational modifications of proteins, special techniques within proteomics are used. Redox proteomics requires special labeling methods, including the reduction and subsequent labeling of the oxidized thiol groups with 5-iodoacetamidofluorescein (IAF) [126]. Twenty-two highly oxidized proteins involved in a wide range of biological processes were identified in ozone-treated rice using this method [127]. Phosphoproteome [128], glycoproteome, and secretome [129–132] are sub-categories of proteomics that require special staining and enrichment techniques. Post-translational modifications involved in gene expression regulations will be discussed in the Epigenomics section below.

### 4.3. Interactome

Protein-protein interactions determine the contextual functions of a protein and hence play a crucial role in regulation and signal transduction [133]. There are several commonly used experimental systems to identify protein-protein interactions, including: (1) yeast two hybrid (Y2H) (reviewed in [134]); (2) biomolecular fluorescence complementation (BiFC) (reviewed in [134]); (3) affinity pull-down coupled with mass spectrometry (AP-MS) (reviewed in [134]); (4) blue native PAGE [135]; and (5) structural analysis of protein crystals [136,137]. In addition, literature curation involving tedious literature searches can be used to supplement the experimental efforts [138] and *in silico* prediction can be done by searching for orthologous pairs which interact in other systems, to identify possible interologues [134,139]. Multiple systems are generally adopted to authenticate the interactions.

The concept of the plant interactome was initiated years ago, and was based mainly on the information collected through literature curation [140]. Subsequently, an experimentally constructed interactome map of *A. thaliana* was established via intensive screening, recording a total of 6200 high-confidence interactions among 2700 proteins through the screening of proteins encoded by 8000 open reading frames in the Arabidopsis genome [141]. It is estimated that this screening only captured around 2% of the binary protein-protein interactome in *A. thaliana* [141]. Using the *in silico* interolog prediction method, more than 37,000 interactions among 4567 rice proteins were predicted, 168 of which have been experimentally confirmed [139]. In this piece of work, the INPARANOID 3.0 program was used to predict high-confidence protein orthologues in 12 species including rice. With the assumption that protein-protein interactions are retained in evolutionarily conserved orthologous proteins, rice protein-protein interactions were compiled using the predicted orthologous proteins and the known interactions in interactome databases [139]. Only a few studies directly related to crop stress interactomes have been published (Table 6). In the search for rice stress-related interactomes, 4 stress proteins related to disease (XA21 and NH1) and flooding (SUB1A and SUB1C) were used as baits for the initial interactome screens by Y2H [142]. Preys identified from the initial screens were then used as baits for subsequent screens. Together with the information from literature curation, an interactome network consisting of 100 proteins were constructed. The interactomes of the two kinds of stresses were linked by proteins such as SNRK1A, which has been shown to be related to ABA, a positive regulator of abiotic stress responses and a negative regulator of biotic stress responses [142].

Online resources such as PRIN [143] can help to predict rice interactomes, while BioGRID [144], DIP [145], PlaPID [146], and InAct [147] can be queried for some pre-determined interactomes in certain plant species. Recent large-scale stress interactome studies in crop plants are shown in Table 6.

### 4.4. Epigenome

In addition to the genetic information encoded by DNA, epigenetic modifications of DNA and histones provide another dimension of regulation to influence gene expressions. Chromatin-associated proteins, including DNA methylase, histones, and histone-modifying enzymes, are cataloged in the ChromDB [151]. Technological platforms for epigenomic research can be considered as an extension of genomic and proteomic studies with modifications in analysis protocols.

For example, cytosine DNA methylation, one of the epigenetic modifications, plays an important role in gene silencing and genomic imprinting [152,153]. The transcriptional levels of endogenous genes are highly correlated with the methylation status within their promoter or transcribed regions [154,155]. One way to detect DNA methylation is to capture and enrich the methylated DNA fragments by immunoprecipitation [156]. Bisulfite treatment is another way to distinguish between methylated and unmethylated DNA. The bisulfite treatment converts unmethylated (but not methylated) cytosines to uracils [157]. Both immunoprecipitation-enriched and bisulfite-treated DNA can be analyzed by microarray- or sequencing-based methods to the single-base level of resolution [157–159]. A number of bioinformatics tools are designed to handle the bisulfite sequencing data (Table 7).

Both biotic and abiotic stresses will lead to massive changes in the DNA methylation status [160–162]. Some stress-induced DNA methylations can be inherited by the next generation. The mechanism for trans-generation DNA methylation may be partially mediated by small RNAs [163]. This trans-generation DNA methylation has been observed in some crops in response to stress [164,165], as a way of pre-acquiring immunity toward the upcoming stresses via designed parental priming [164].

Histone proteins are responsible for the packing of DNA. The epigenetic modifications of core histones affecting the tightness of DNA packing are called histone codes that can relay important information to affect gene expressions [177]. The Histone Sequence Database provides a comprehensive collection of histone sequences and structural information [178].

The addition of acetyl groups to histones neutralizes the positive charges and hence loosens the condensed DNA, leading to transcriptional activation [179], while the methylation of histones results in gene deactivation or repression [180]. The phosphorylation of histones causes the relaxation of chromatin and modulates histone acetylation and methylation [181].

Individual types of histone modifications on specific amino acid residues can be detected using specific antibodies or various mass spectrometries while genome-wide histone-DNA associations can be captured by chromatin immunoprecipitation (ChIP) and subsequently analyzed using either microarray (ChIP-Chip) [182] or sequencing (ChIP-seq) [183].

Some histone-modifying enzymes are induced in crops under stress. For example, a trithorax-like H3K4 methyltransferase was found to be induced by drought in drought-tolerant barley cultivars [184] while a histone deacetylase was found to be induced by compatible infections and repressed by incompatible infections [185]. The methylation statuses of four transcription factors were affected by salt stress. The expression of three of these transcription factors were also found to be correlated with their H3 methylation and acetylation statuses [186]. A genome-wide study in rice identified 4837 genes that harbor differential H3K4me3 modification under drought stress, in which the expression of 609 genes were significantly correlated with the H3K4me3 modification [187].

### 4.5. Phenome

Every observable biological characteristic beyond the genotype can be regarded as the phenotype. Phenotypes can be observed at the molecular, cellular, organismal, and population levels. Phenotypes also vary throughout the organism’s lifecycle, spanning different growth stages, and during different periods of stress. The environment can also exert significant influences on the phenotype. The total sum of phenotypes of an organism or a population constitutes the phenome.

As mentioned in section 2.2, to make phenotypic data in public databases more searchable and accessible to users of bioinformatics tools, ontologies are used to describe the setup of the experiment and the phenotypic data. For example, one may study salt tolerance (TO:0006001) at the whole-plant flowering stage (PO:0007016) and the days to flower (TO:0000344) of *Oryza sativa* (GR_tax:013681), in a greenhouse study (EO:0007248) under a sodium chloride regimen (EO:0007048). These ontologies provide a common language to describe an experiment and render it understandable by both researchers and computational algorithms. For instance, some people may record certain phenotypes during the flowering stage. However, what does it mean by “flowering stage”? Some people refer to “flowering stage” as the time when the first flower opens. Others may refer to it as having half of the individual plants with flowers opened. In this case, the flowering time is well defined in plant ontology. PO:0007026, PO:0007034, PO:0007053 and PO:0007052 refer to the stage at which the first flower, 1/4 of the flowers, 1/2 of the flowers, and 3/4 of the flowers, open, respectively. PO:0007024 marks the end of the flowering stage. The application of these ontologies can thus reduce the discrepancies in annotating the phenotypes and treatment conditions.

High-quality phenotypic information is essential for mapping, association studies, gene identifications, gene functional studies and genomic selections. To design experiments to collect phenotypic information, some critical parameters have to be considered, such as the sample/population sizes, experimental conditions, phenotypes to be assessed, and the data acquisition methods.

The size of a population can vary from a few plants for functional studies, several hundred lines for mapping and GS, to as many as a thousand germplasms for GWAS. Some public collections of germplasms or populations are available for public requests. The United States Department of Agriculture National Plant Germplasm System has a collection of over 500,000 germplasm accessions from 10,000 plant species including rice, soybean, tomato and many other staple crops. Table 8 summarized some publicly available mutant and germplasm collections, some of which also provide phenotypic descriptions and photos of the mutant.

Since the phenome is the overall outcome of the interactions between the genotype and the environment, whether the phenotypic data are collected in a controlled environment or not can greatly affect the final interpretation of results. Field experiments can better mimic the actual conditions of crop production, but the consistency of the phenotype greatly depends on the location of the field, the soil composition, weather conditions, season, and so on. The interpretation of results can thus be complicated. For example, a change in the transpiration rate in some of the plants may not solely be the result of the stress treatment, but also the result of localized changes in light intensity and/or temperature in the field [188]. In general, a larger number of replications are required to compensate for the effects due to environmental variations. A controlled environment such as that in a greenhouse or a growth chamber can minimize the effects of environmental fluctuations and hence will emphasize the contribution of the genotype. However, data from controlled experiments are usually limited in scale and may overlook the fast-changing environment in the real production field.

Choosing the appropriate phenotypes to be assessed is also important. For example, stomatal conductance and pathogen titer are good indicators of osmotic stress tolerance and disease resistance, respectively. However, they are not quite applicable in large-scale experiments due to the limitation of the machine and the laborious procedures. On the other hand, fresh weight and biomass can truly reflect the productivity of the crops, but taking these measurements is destructive to the plant. For morphological and physiological phenotyping of crops under stress, a conversion of stress symptoms to parameters that can be captured and digitized is needed for high-throughput automation. Commonly used methods include: 2D or 3D visible light imaging [189,190], infrared thermography [188], near-infrared imaging, spectral reflectance [191], fluorescence analysis [191,192], stable isotope analysis [193] and X-ray imaging [194]. For example, a study of wheat salt stress response suggested that the shoot area calculated from 3 digital images (2 side and 1 top images) showed a strong positive correlation with manually measured leaf area and shoot fresh weight which commonly serve as the indicators of salt tolerance in crops [195]. As a non-destructive method, the imaging system could continuously monitor the growth of the plant and distinguish its bi-phasic (osmotic stress phase and ionic stress phase) growth under salinity stress [195]. Another example is related to osmotic stresses (salinity and drought) that reduce stomatal conductance. Since the reduction in stomatal conductance will halt the cooling effect of transpiration, infrared thermal imaging can be used to monitor the degree of salinity and drought stress [188,196]. In the case of lesions on leaf surfaces caused by plant diseases, instead of measuring the lesion area on each leaf, determining the reduction in chlorophyll fluorescence is a possible alternative [197].

In addition to the physiological phenotypes, metabolite profiles in crops are also altered by both biotic and abiotic stresses [198,199]. Deleterious metabolites such as reactive oxygen species might be generated through the disruption of normal cellular processes while beneficial metabolites such as signaling molecules and osmoprotectants may be generated to alleviate the stress [200,201].

Current platforms for metabolomic analyses include various forms of liquid chromatography-coupled mass spectrometry (LC-MS), gas chromatography-coupled MS (GC-MS), capillary electrophoresis-coupled MS (CE-MS), fourier transform MS (FT-MS), fourier transform infrared spectrometry (FT-IR), and one- or two-dimension nuclear magnetic resonance (NMR). Raw spectra generated by mass spectrometry or NMR can be analyzed by making references to either in-house or online databases (Table 9). Biological multivariate data generated from metabolomic studies are commonly analyzed using principal component analysis (PCA) [202], partial least square (PLS) [203], and orthogonal projections to latent structures (O-PLS) [203,204].

Numerous metabolomic studies have been done on crops under stress, including: salinity [215–217], drought [218–221], flooding [222], ozone treatment [223], fungal infections [224–226], bacterial infections [217,227], other infections [228], and multiple stresses [229,230].

There are two major research strategies in metabolomic studies: metabolic fingerprinting and metabolic profiling. Metabolic fingerprinting uses the mass-to-charge ratio of mass spectrometry, the peak height and/or retention time of chromatography and the strength of NMR signal as the metabolomic signature in specific samples, such that the identity of each metabolite is not necessary [231]. This helps to classify different samples into categories. For example, metabolic fingerprints have been made to differentiate between disease-resistant and susceptible varieties [217,227,228] or between salt-tolerant and sensitive varieties [232]. In one study, fourier transform infrared (FT-IR) spectroscopy was used for the metabolic fingerprinting of salt-treated tomatoes [233]. A total of 882 FT-IR spectra variables were collected between the wave number 4000 to 600 cm^−1^ for each sample [233]. Through discriminant function analysis (DFA) of the spectra variables, without knowing the identity and the quantity of each metabolite, salt-treated and control samples can be discriminated. Furthermore, key regions within the spectrum distinguishing the treated from the untreated samples were identified through genetic algorithms, and the major components were found to be amino radicals and nitrile-containing compounds [233]. Thus, disease resistance and stress tolerance of novel crop varieties can be assessed by comparing their metabolic fingerprints with those of well characterized varieties, facilitating the screening process.

On the other hand, metabolic profiling compares the metabolic compositions between samples and hence the quantitation and identification of the metabolites are required. Signal patterns must be matched to known standards or depositions in the databases in order to identify the actual compounds. For example, the accumulation of compatible solutes, such as proline, glycine-betaine, and their precursors, is usually observed in osmotically stressed crops, especially in tolerant varieties [216,220,221]. A specific example is the mitochondrial metabolic profile of flood-stressed soybean; metabolites were extracted from the roots and hypocotyls of soybean seedlings with or without submergence stress [226], and were then analyzed using capillary electrophoresis mass spectrometry. Eighty-one mitochondria-related metabolites were identified and quantified with reference to the commercially available standards [226]. There was an accumulation of TCA cycle-related metabolites, including citrate, succinate, and aconitate, but a reduction in ATP in flood-stressed plants, which can be explained by the arrest of aerobic respiration due to anoxia [222]. Following a similar logic, the accumulation of antimicrobial compounds, such as caffeic acid, phytoalexins, glycoalkaloids, and other polyphenolic compounds, are common in pathogen-infected crops compared to their uninfected counterparts [224–226,228]. Glucose oxidase secreted by a fungal pathogen, *Botrytis cinerea*, can also lead to the accumulation of gluconic acid in *Vitis vinifera* cv. Chardonnay berries [226].

## 5. Future Perspectives

Sequencing throughput is no longer the major limiting factor in genomics and transcriptomics studies. The next generation sequencing platforms can actually generate enough depth for genome assembly in one or several runs [234]. However, sequence assembly and annotation for complex genomes remain challenging. The data acquisition platforms for other “-omics”, on the other hand, are under rapid development to catch up with the pace of genomic research. While the data source is no longer a rate-determining step, data integration and interpretation have become the bottleneck in the research pipeline. One obstacle hindering the cross-platform analyses of different datasets is the variations in experimental designs, treatment conditions, and data formats. Drawing meaningful conclusions may sometimes be difficult when there are discrepancies between two germplasms. For example, the transcriptomic data of one germplasm may not be used effectively to explain the proteomic data of another germplasm. Researchers should therefore strategically design experiments to generate interrelated -omics data using carefully selected germplasms. The standardization of data acquisition and storage formats using strictly controlled vocabulary is also important.

With the advance of computer technology and high-throughput analysis platforms, life processes can now be captured, digitized, and stored in the hard disk of a computer. Yet, no matter how perfectly a genome is sequenced and assembled, biological data from experiments are still essential to connect the genotypes and the phenotypes. A few softwares/platforms have been developed to integrate the interactions of cellular components into networks [235,236]. For example, the VirtualPlant has been developed as a software platform for the integration and analysis of different levels of data [237]. It provides large datasets of Arabidopsis gene annotation, gene functional categories, microarray data, biochemical pathways, interaction information, and microRNA:mRNA interaction information. Users can also upload their own gene lists and microarray data for analysis, and identify coexpressed genes, interacting proteins, and metabolites associated with their genes of interest. Building a similar platform for crop plants could be extremely useful but it requires a well-coordinated effort among different research centers and groups.

## Supplementary Information



## Figures and Tables

**Table 1:** Example of some commonly used databases.

Database name	URL	Reference
GenBank	http://www.ncbi.nlm.nih.gov/genbank/	[4]
ENA	http://www.ebi.ac.uk/ena/	[5]
DDBJ	http://www.ddbj.nig.ac.jp/	[6]
Phytozome	http://www.phytozome.net/	[7]
Gramene	http://www.gramene.org/	[8]
KEGG	http://www.genome.jp/kegg/	[9]
PlantGDB	http://www.plantgdb.org/	[10]
EnsemblPlants	http://plants.ensembl.org/index.html	[11]
VISTA	http://genome.lbl.gov/vista/index.shtml	[12]
PLAZA	http://bioinformatics.psb.ugent.be/plaza/	[13]
GigaDB	http://gigadb.org/	[14]
SGN	http://solgenomics.net/	[15]
GrainGenes	http://wheat.pw.usda.gov	[16]
LIS	http://www.comparative-legumes.org/	[17]

**Table 2:** Data repositories for crop plants.

Crop	Database name	URL of related database	Ref
Rice	RAP-DB	http://rapdb.dna.affrc.go.jp/	[23]
Maize	MaizeGDB	http://www.maizegdb.org/	[24]
Medicago	*Medicago truncatula* SEQUENCING RESOURCE	http://www.medicago.org/genome/index_old.php	-
Wheat	GrainGenes	http://wheat.pw.usda.gov/	[16]
Potato	Solanaceae Genomics Resource	http://solanaceae.plantbiology.msu.edu/index.shtml	-
Soybean	SoyBase	http://soybase.org	[25]
Tomato	TOMATO FUNCTIONAL GENOMICS DATABASE	http://ted.bti.cornell.edu/	[26]

**Table 3:** List of ontologies containing information related to crop stress responses.

Domain	Prefix	Description	Reference/website
Plant Environmental Conditions	EO	Controlled vocabulary for the representation of plant environmental conditions	http://www.gramene.org/db/ontology/search?id=EO:0007359
Gene Ontology	GO	Controlled vocabulary for genes and gene products	[28]
Taxonomy Ontology	GR_tax	Representation of the taxonomic tree of plants in the ontology format	http://www.gramene.org/db/ontology/search?id=GR_tax:090165
The Plant-Associated Microbe Gene Ontology	PAMGO	Controlled vocabulary for the interaction of microbes with their hosts	[29]
Plant Ontology	PO	Controlled vocabulary for anatomy, morphology and stages of development for all plants	[30]
Sequence Ontology	SO	Controlled vocabulary for sequence annotations, for the exchange of annotation data and for the description of sequence objects in databases	[31]
Plant Trait Ontology	TO	Controlled vocabulary for phenotypic traits in plants	http://www.gramene.org/db/ontology/search?id=TO:0000387

**Table 4:** Widely used bioinformatics tools for the analysis of transcriptome data.

Software	Description	Download URL	Reference
ABMapper	RNA-seq data alignment	http://hkbic.cuhk.edu.hk/software/abmapper	[91]
Bowtie	RNA-seq data alignment	http://bowtie-io.sourceforge.net/bowtie2/index.shtml	[92]
Cufflinks	Transcript assembly	http://cufflinks.cbcb.umd.edu/	[93]
DEGseq	Differential gene expression detection	http://www.bioconductor.org/packages/2.11/bioc/html/DEGseq.html	[94]
Infernal	RNA-seq data alignment	http://infernal.janelia.org/	[95]
Oases	*De novo* assembly	www.ebi.ac.uk/~zerbino/oases/	[96]
Tophat	RNA-seq data alignment & Alternative splicing detection	http://tophat.cbcb.umd.edu/	[97]
Trans-AByss	*De novo* assembly	http://www.bcgsc.ca/platform/bioinfo/software/	[98]
Trinity	*De novo* assembly	http://trinityrnaseq.sourceforge.net/	[99]

**Table 5:** Bioinformatics resources commonly used in crop proteomic studies.

Program	URL
Mascot	http://www.matrixscience.com
SEQUEST	http://fields.scripps.edu/sequest/index.html
X!Tandem	http://www.thegpm.org/TANDEM/index.html

**Database**	**URL**

Expasy	http://www.uniprot.org/help/uniprotkb
UniprotKB/SwissProt and UniProtKB/TrEMBL database	http://www.uniprot.org/help/uniprotkb
Protein Information Resource (PIR)	http://pir.georgetown.edu/
RCSB Protein Data Bank (RCSB PDB)	http://www.rcsb.org/pdb/download/download.do
EMBL-EBI’s Protein Data Bank in Europe (PDBe)	http://www.ebi.ac.uk/pdbe/
SWISS-2DPAGE	http://world-2dpage.expasy.org/swiss-2dpage/
The Plant Proteome Database (PPDB)	http://ppdb.tc.cornell.edu/
Plant Protein Phosphorylation DataBase (P^3^DB)	http://www.p3db.org/
RIKEN Plant Phosphoproteome Database (RIPP)	https://database.riken.jp/sw/links/en/ria102i/
Secretom—The Tomato Fruit Glycoproteome	http://solgenomics.net/secretom/detail/glycoproteome
Plant Secretome KnowledgeBase (PlantSecKB)	http://proteomics.ysu.edu/secretomes/plant.php

**Table 6:** Recent large-scale stress interactome studies in crop plants.

Species	Stress	Strategy	Reference
Rice	Abiotic and biotic	Using stress components as bait in Y2H	[142,148]
Wheat	Cold and dehydration	Using stress components as bait in Y2H	[149]
Soybean	SCN infection	*In silico* prediction	[150]

**Table 7:** Bioinformatics tools for the analysis of bisulfite sequencing data.

Tools	Descriptions	Reference
BiQ Analyzer HT	Quantitative study of locus-specific DNA methylation patterns from bisulfite sequencing data.	[166]
Bismark	Mapping of bisulfite-sequencing reads and methylation calling.	[167]
BRAT-BW	Genome-wide single base-resolution methylation data analysis	[168]
BSmooth	Providing estimate of methylation profiles with low-coverage whole-genome bisulfite-sequencing data.	[169]
BS seeker	Mapping of bisulfite-sequencing reads.	[170]
BSMAP	Bisulfite reads mapping algorithm.	[171]
CpG_MPs	Analysis of biosulfite-sequencing read and identification of genome-wide methylation pattern	[172]
GBSA	Both gene-centric or gene-independent analyses of whole-genome bisulfite sequencing data	[173]
Kismeth	Analysis of plant bisulfite sequencing results, with a tool for designing bisulfite sequencing primers.	[174]
QUMA	Quantification tool for methylation analysis	[175]
RRBSMAP	Derivative of BSMAP—a specific tool for reduced-representation bisulfite sequencing	[176]

**Table 8:** Databases for mutant and germplasm resources.

Species	Databases	URL
Barley	Barley DB	http://www.shigen.nig.ac.jp/barley/
Barley	NordGen Plant Collection	http://www.nordgen.org
Maize	RescueMu Maize Mutant Phenotype Database	http://maizegdb.org/rescuemu-phenotype.php
Rice	Oryza Tag Line	http://oryzatagline.cirad.fr/
Rice	Rice Tos17 Insertion Mutant Database	http://pfg101.nias.affrc.go.jp/
Soybean	SoyBase—Fast Neutron Mutants	http://www.soybase.org/mutants/index.php
Tomato	Genes that Make Tomatoes	http://zamir.sgn.cornell.edu/mutants/index.html
Tomato	LycoTILL	http://www.agrobios.it/tilling/index.html
Tomato	TOMATOMA	http://tomatoma.nbrp.jp/index.jsp
Tomato/Pea	URGV TILLING Database	http://urgv.evry.inra.fr/UTILLdb
Tomato/Potato	SOL genomics network	http://solgenomics.net/
Wheat	The Scottish Wheat Variety Database	http://wheat.agricrops.org/menu.php

**Table 9:** Mass spectra databases and other bioinformatics resources for metabolomic studies.

Database	URL	Reference
AOCS Lipid Library	http://lipidlibrary.aocs.org/index.html	-
Golm Metabolome Database	http://gmd.mpimp-golm.mpg.de/	[205]
Lipidomics Gateway	http://www.lipidmaps.org/	[206]
Madison-Qingdao Metabolomics Consortium Database	http://mmcd.nmrfam.wisc.edu/	[207]
Manchester Metabolomics Database	http://dbkgroup.org/MMD/	[208]
MassBank	http://www.massbank.jp/index.html	[209]
Metabolome Express	https://www.metabolome-express.org/	[210]
METLIN	http://metlin.scripps.edu/	[211]
NIST Chemistry WebBook	http://webbook.nist.gov/chemistry/#Notes	-

**Software/Tools**	**URL**	**Reference**

AMDIS	http://www.amdis.net/	-
COLMAR	http://spinportal.magnet.fsu.edu/	[212]
MetDat	http://smbl.nus.edu.sg/METDAT2/	[213]
MetaboSearch	http://omics.georgetown.edu/MetaboSearch.html	[214]

## References

[1] FAO (2009). How to Feed the World in 2050.

[2] *Arabidopsis* Genome Initiative (2000). The *Arabidopsis* genome initiative analysis of the genome sequence of the flowering plant *Arabidopsis thaliana*. Nature.

[3] BGI Library of Digital Life: Plants.

[4] Sayers E.W., Barrett T., Benson D.A., Bolton E., Bryant S.H., Canese K., Chetvernin V., Church D.M., DiCuccio M., Federhen S. (2012). Database resources of the National Center for Biotechnology Information. Nucleic Acids Res.

[5] Leinonen R., Akhtar R., Birney E., Bower L., Cerdeno-Tárraga A., Cheng Y., Cleland I., Faruque N., Goodgame N., Gibson R. (2011). The European nucleotide archive. Nucleic Acids Res.

[6] Miyazaki S., Sugawara H., Ikeo K., Gojobori T., Tateno Y. (2004). DDBJ in the stream of various biological data. Nucleic Acids Res.

[7] Goodstein D.M., Shu S., Howson R., Neupane R., Hayes R.D., Fazo J., Mitros T., Dirks W., Hellsten U., Putnam N. (2012). Phytozome: A comparative platform for green plant genomics. Nucleic Acids Res.

[8] Liang C., Jaiswal P., Hebbard C., Avraham S., Buckler E.S., Casstevens T., Hurwitz B., McCouch S., Ni J., Pujar A. (2008). Gramene: A growing plant comparative genomics resource. Nucleic Acids Res.

[9] Kanehisa M., Araki M., Goto S., Hattori M., Hirakawa M., Itoh M., Katayama T., Kawashima S., Okuda S., Tokimatsu T. (2008). KEGG for linking genomes to life and the environment. Nucleic Acids Res.

[10] Duvick J., Fu A., Muppirala U., Sabharwal M., Wilkerson M.D., Lawrence C.J., Lushbough C., Brendel V. (2008). PlantGDB: A resource for comparative plant genomics. Nucleic Acids Res.

[11] Kersey P.J., Staines D.M., Lawson D., Kulesha E., Derwent P., Humphrey J.C., Hughes D.S.T., Keenan S., Kerhornou A., Koscielny G. (2012). Ensembl Genomes: An integrative resource for genome-scale data from non-vertebrate species. Nucleic Acids Res.

[12] Frazer K.A., Pachter L., Poliakov A., Rubin E.M., Dubchak I. (2004). VISTA: Computational tools for comparative genomics. Nucleic Acids Res.

[13] Proost S., van Bel M., Sterck L., Billiau K., van Parys T., van de Peer Y., Vandepoele K. (2009). PLAZA: A comparative genomics resource to study gene and genome evolution in plants. Plant Cell.

[14] Sneddon T.P., Li P., Edmunds S.C. (2012). GigaDB: Announcing the GigaScience database. GigaScience.

[15] Bombarely A., Menda N., Tecle I.Y., Buels R.M., Strickler S., Fischer-York T., Pujar A., Leto J., Gosselin J., Mueller L.A. (2011). The Sol Genomics Network (solgenomics.net): Growing tomatoes using Perl. Nucleic Acids Res.

[16] Carollo V., Matthews D.E., Lazo G.R., Blake T.K., Hummel D.D., Lui N., Hane D.L., Anderson O.D. (2005). GrainGenes 2.0. An improved resource for the small-grains community. Plant Physiol.

[17] Gonzales M.D., Archuleta E., Farmer A., Gajendran K., Grant D., Shoemaker R., Beavis W.D., Waugh M.E. (2005). The Legume Information System (LIS): An integrated information resource for comparative legume biology. Nucleic Acids Res.

[18] Paterson A.H., Bowers J.E., Bruggmann R., Dubchak I., Grimwood J., Gundlach H., Haberer G., Hellsten U., Mitros T., Poliakov A. (2009). The *Sorghum bicolor* genome and the diversification of grasses. Nature.

[19] The International Barley Genome Sequencing Consortium (2012). A physical, genetic and functional sequence assembly of the barley genome. Nature.

[20] Zhang G., Liu X., Quan Z., Cheng S., Xu X., Pan S., Xie M., Zeng P., Yue Z., Wang W. (2012). Genome sequence of foxtail millet (*Setaria italica*) provides insights into grass evolution and biofuel potential. Nat. Biotech.

[21] Messmer R., Fracheboud Y., Bänziger M., Vargas M., Stamp P., Ribaut J.-M. (2009). Drought stress and tropical maize: QTL-by-environment interactions and stability of QTLs across environments for yield components and secondary traits. Theor. Appl. Genet.

[22] Sanseverino W., Hermoso A., D’Alessandro R., Vlasova A., Andolfo G., Frusciante L., Lowy E., Roma G., Ercolano M.R. (2013). PRGdb 2.0: Towards a community-based database model for the analysis of R-genes in plants. Nucleic Acids Res.

[23] Sakai H., Lee S.S., Tanaka T., Numa H., Kim J., Kawahara Y., Wakimoto H., Yang C.-C., Iwamoto M., Abe T. (2013). Rice annotation project database (RAP-DB): An integrative and interactive database for rice genomics. Plant Cell Physiol.

[24] Schaeffer M.L., Harper L.C., Gardiner J.M., Andorf C.M., Campbell D.A., Cannon E.K.S., Sen T.Z., Lawrence C.J. (2011). MaizeGDB: Curation and outreach go hand-in-hand. Database.

[25] Grant D., Nelson R.T., Cannon S.B., Shoemaker R.C. (2010). SoyBase, the USDA-ARS soybean genetics and genomics database. Nucleic Acids Res.

[26] Fei Z., Joung J.-G., Tang X., Zheng Y., Huang M., Lee J.M., McQuinn R., Tieman D.M., Alba R., Klee H.J. (2011). Tomato functional genomics database: A comprehensive resource and analysis package for tomato functional genomics. Nucleic Acids Res.

[27] Gene Ontology Consortium The Gene Ontology Website.

[28] Ashburner M., Ball C.A., Blake J.A., Botstein D., Butler H., Cherry J.M., Davis A.P., Dolinski K., Dwight S.S., Eppig J.T. (2000). Gene Ontology: Tool for the unification of biology. Nat. Genet.

[29] Torto-Alalibo T., Collmer C., Gwinn-Giglio M. (2009). The Plant-Associated Microbe Gene Ontology (PAMGO) Consortium: Community development of new Gene Ontology terms describing biological processes involved in microbe-host interactions. BMC Microbiol.

[30] Avraham S., Tung C.W., Ilic K., Jaiswal P., Kellogg E.A., McCouch S., Pujar A., Reiser L., Rhee S.Y., Sachs M.M. (2008). The Plant Ontology Database: A community resource for plant structure and developmental stages controlled vocabulary and annotations. Nucleic Acids Res.

[31] Eilbeck K., Lewis S.E., Mungall C.J., Yandell M., Stein L., Durbin R., Ashburner M. (2005). The Sequence Ontology: A tool for the unification of genome annotations. Genome Biol.

[32] The Catalogue of Life.

[33] Integrated Taxonomic Information System http://www.itis.gov/.

[34] Wang K., Wang Z., Li F., Ye W., Wang J., Song G., Yue Z., Cong L., Shang H., Zhu S. (2012). The draft genome of a diploid cotton *Gossypium raimondii*. Nat. Genet.

[35] Shulaev V., Korban S.S., Sosinski B., Abbott A.G., Aldwinckle H.S., Folta K.M., Iezzoni A., Main D., Arús P., Dandekar A.M. (2008). Multiple models for rosaceae genomics. Plant Physiol.

[36] Shulaev V., Sargent D.J., Crowhurst R.N., Mockler T.C., Folkerts O., Delcher A.L., Jaiswal P., Mockaitis K., Liston A., Mane S.P. (2011). The genome of woodland strawberry (*Fragaria vesca*). Nat. Genet.

[37] Brenchley R., Spannagl M., Pfeifer M., Barker G.L.A., D’Amore R., Allen A.M., McKenzie N., Kramer M., Kerhornou A., Bolser D. (2012). Analysis of the bread wheat genome using whole-genome shotgun sequencing. Nature.

[38] Jia J., Zhao S., Kong X., Li Y., Zhao G., He W., Appels R., Pfeifer M., Tao Y., Zhang X. (2013). *Aegilops tauschii* draft genome sequence reveals a gene repertoire for wheat adaptation. Nature.

[39] Ling H.-Q., Zhao S., Liu D., Wang J., Sun H., Zhang C., Fan H., Li D., Dong L., Tao Y. (2013). Draft genome of the wheat A-genome progenitor *Triticum urartu*. Nature.

[40] Lam H.M., Xu X., Liu X., Chen W.B., Yang G.H., Wong F.L., Li M.W., He W.M., Qin N., Wang B. (2010). Resequencing of 31 wild and cultivated soybean genomes identifies patterns of genetic diversity and selection. Nat. Genet.

[41] Xu X., Liu X., Ge S., Jensen J.D., Hu F., Li X., Dong Y., Gutenkunst R.N., Fang L., Huang L. (2012). Resequencing 50 accessions of cultivated and wild rice yields markers for identifying agronomically important genes. Nat. Biotechnol.

[42] Hufford M.B., Xu X., van Heerwaarden J., Pyhajarvi T., Chia J.-M., Cartwright R.A., Elshire R.J., Glaubitz J.C., Guill K.E., Kaeppler S.M. (2012). Comparative population genomics of maize domestication and improvement. Nat. Genet.

[43] Kim M.Y., Lee S., Van K., Kim T.-H., Jeong S.-C., Choi I.-Y., Kim D.-S., Lee Y.-S., Park D., Ma J. (2010). Whole-genome sequencing and intensive analysis of the undomesticated soybean (*Glycine soja* Sieb. and Zucc.) genome. Proc. Natl. Acad. Sci. USA.

[44] Lai J., Li R., Xu X., Jin W., Xu M., Zhao H., Xiang Z., Song W., Ying K., Zhang M. (2010). Genome-wide patterns of genetic variation among elite maize inbred lines. Nat. Genet.

[45] Garcia-Mas J., Benjak A., Sanseverino W., Bourgeois M., Mir G., Gonzalez V.M., Henaff E., Camara F., Cozzuto L., Lowy E. (2012). The genome of melon (*Cucumis melo* L.). Proc. Natl. Acad. Sci. USA.

[46] Dangl J.L., Jones J.D.G. (2001). Plant pathogens and integrated defence responses to infection. Nature.

[47] Shanmugam V. (2005). Role of extracytoplasmic leucine rich repeat proteins in plant defence mechanisms. Microbiol. Res.

[48] Torii K.U. (2004). Leucine-rich repeat receptor kinases in plants: Structure, function, and signal transduction pathways. Int. Rev. Cytol.

[49] Van Ooijen G., Mayr G., Kasiem M.M.A., Albrecht M., Cornelissen B.J.C., Takken F.L.W. (2008). Structure—Function analysis of the NB-ARC domain of plant disease resistance proteins. J. Exp. Bot.

[50] McNally K.L., Childs K.L., Bohnert R., Davidson R.M., Zhao K., Ulat V.J., Zeller G., Clark R.M., Hoen D.R., Bureau T.E. (2009). Genomewide SNP variation reveals relationships among landraces and modern varieties of rice. Proc. Natl. Acad. Sci. USA.

[51] Zheng L.-Y., Guo X.-S., He B., Sun L.-J., Peng Y., Dong S.-S., Liu T.-F., Jiang S., Ramachandran S., Liu C.-M. (2011). Genome-wide patterns of genetic variation in sweet and grain sorghum (*Sorghum bicolor*). Genome Biol.

[52] Dodds P.N., Rathjen J.P. (2010). Plant immunity: Towards an integrated view of plant-pathogen interactions. Nat. Rev. Genet.

[53] D’Hont A., Denoeud F., Aury J.-M., Baurens F.-C., Carreel F., Garsmeur O., Noel B., Bocs S., Droc G., Rouard M. (2012). The banana (*Musa acuminata*) genome and the evolution of monocotyledonous plants. Nature.

[54] Singh K.B., Foley R.C., Oñate-Sánchez L. (2002). Transcription factors in plant defense and stress responses. Curr. Opin. Plant Biol.

[55] Collard B.C.Y., Jahufer M.Z.Z., Brouwer J.B., Pang E.C.K. (2005). An introduction to markers, quantitative trait loci (QTL) mapping and marker-assisted selection for crop improvement: The basic concepts. Euphytica.

[56] Rafalski J.A. (2010). Association genetics in crop improvement. Curr. Opin. Plant Biol.

[57] Huang S., Spielmeyer W., Lagudah E.S., James R.A., Platten J.D., Dennis E.S., Munns R. (2006). A sodium transporter (HKT7) is a candidate for *Nax1*, a gene for salt tolerance in durum wheat. Plant Physiol.

[58] Ren Z.-H., Gao J.-P., Li L.-G., Cai X.-L., Huang W., Chao D.-Y., Zhu M.-Z., Wang Z.-Y., Luan S., Lin H.-X. (2005). A rice quantitative trait locus for salt tolerance encodes a sodium transporter. Nat. Genet.

[59] Sutton T., Baumann U., Hayes J., Collins N.C., Shi B.-J., Schnurbusch T., Hay A., Mayo G., Pallotta M., Tester M. (2007). Boron-toxicity tolerance in barley arising from efflux transporter amplification. Science.

[60] Huang X., Feng Q., Qian Q., Zhao Q., Wang L., Wang A., Guan J., Fan D., Weng Q., Huang T. (2009). High-throughput genotyping by whole-genome resequencing. Genome Res.

[61] Huang X., Wei X., Sang T., Zhao Q., Feng Q., Zhao Y., Li C., Zhu C., Lu T., Zhang Z. (2010). Genome-wide association studies of 14 agronomic traits in rice landraces. Nat. Genet.

[62] Yu H., Xie W., Wang J., Xing Y., Xu C., Li X., Xiao J., Zhang Q. (2011). Gains in QTL detection using an ultra-high density SNP map based on population sequencing relative to traditional RFLP/SSR markers. PLoS One.

[63] Zou G., Zhai G., Feng Q., Yan S., Wang A., Zhao Q., Shao J., Zhang Z., Zou J., Han B. (2012). Identification of QTLs for eight agronomically important traits using an ultra-high-density map based on SNPs generated from high-throughput sequencing in sorghum under contrasting photoperiods. J. Exp. Bot.

[64] Li H., Durbin R. (2010). Fast and accurate long-read alignment with Burrows–Wheeler transform. Bioinformatics.

[65] Wetterstrand K. DNA Sequencing Costs: Data from the NHGRI Genome Sequencing Program (GSP).

[66] Heffner E.L., Sorrells M.E., Jannink J.-L. (2009). Genomic selection for crop improvement. Crop Sci.

[67] Meuwissen T.H.E., Hayes B.J., Goddard M.E. (2001). Prediction of total genetic value using genome-wide dense marker maps. Genetics.

[68] Zhao Y., Gowda M., Liu W., Würschum T., Maurer H.P., Longin F.H., Ranc N., Reif J.C. (2012). Accuracy of genomic selection in European maize elite breeding populations. Theor. Appl. Genet.

[69] Heffner E.L., Jannink J.-L., Iwata H., Souza E., Sorrells M.E. (2011). Genomic selection accuracy for grain quality traits in biparental wheat populations. Crop Sci.

[70] Heffner E.L., Jannink J.-L., Sorrells M.E. (2011). Genomic selection accuracy using multifamily prediction models in a wheat breeding program. Plant Gen.

[71] Zhong S.Q., Dekkers J.C.M., Fernando R.L., Jannink J.L. (2009). Factors affecting accuracy from genomic selection in populations derived from multiple inbred lines: A barley case study. Genetics.

[72] Oliveira E., Resende M., Silva Santos V., Ferreira C., Oliveira G., Silva M., Oliveira L., Aguilar-Vildoso C. (2012). Genome-wide selection in cassava. Euphytica.

[73] Xu S. (2003). Estimating polygenic effects using markers of the entire genome. Genetics.

[74] Nakaya A., Isobe S.N. (2012). Will genomic selection be a practical method for plant breeding?. Ann. Bot.

[75] Jannink J.-L., Lorenz A.J., Iwata H. (2010). Genomic selection in plant breeding: From theory to practice. Brief. Funct. Genomics.

[76] Rutkoski J., Heffner E., Sorrells M. (2011). Genomic selection for durable stem rust resistance in wheat. Euphytica.

[77] Salse J. (2012). *In silico* archeogenomics unveils modern plant genome organisation, regulation and evolution. Curr. Opin. Plant Biol.

[78] Salamov A.A., Solovyev V.V. (2000). *Ab initio* gene finding in drosophila genomic DNA. Genome Res.

[79] Howe K.L., Chothia T., Durbin R. (2002). GAZE: A generic framework for the integration of gene-prediction data by dynamic programming. Genome Res.

[80] Allen J.E., Salzberg S.L. (2005). JIGSAW: Integration of multiple sources of evidence for gene prediction. Bioinformatics.

[81] Finn R.D., Mistry J., Tate J., Coggill P., Heger A., Pollington J.E., Gavin O.L., Gunasekaran P., Ceric G., Forslund K. (2010). The Pfam protein families database. Nucleic Acids Res.

[82] Hunter S., Jones P., Mitchell A., Apweiler R., Attwood T.K., Bateman A., Bernard T., Binns D., Bork P., Burge S. (2012). InterPro in 2011: New developments in the family and domain prediction database. Nucleic Acids Res.

[83] Kanehisa M., Goto S., Sato Y., Furumichi M., Tanabe M. (2012). KEGG for integration and interpretation of large-scale molecular data sets. Nucleic Acids Res.

[84] Altschul S.F., Gish W., Miller W., Myers E.W., Lipman D.J. (1990). Basic local alignment search tool. J. Mol. Biol.

[85] Letunic I., Doerks T., Bork P. (2012). SMART 7: Recent updates to the protein domain annotation resource. Nucleic Acids Res.

[86] Finn R.D., Clements J., Eddy S.R. (2011). HMMER web server: Interactive sequence similarity searching. Nucleic Acids Res.

[87] Sharoni A.M., Nuruzzaman M., Satoh K., Shimizu T., Kondoh H., Sasaya T., Choi I.-R., Omura T., Kikuchi S. (2011). Gene structures, classification and expression models of the AP2/EREBP transcription factor family in rice. Plant Cell Physiol.

[88] Schena M., Shalon D., Davis R.W., Brown P.O. (1995). Quantitative monitoring of gene expression patterns with a complementary DNA microarray. Science.

[89] Ozsolak F., Milos P.M. (2011). RNA sequencing: Advances, challenges and opportunities. Nat. Rev. Genet.

[90] Rustici G., Kolesnikov N., Brandizi M., Burdett T., Dylag M., Emam I., Farne A., Hastings E., Ison J., Keays M. (2013). ArrayExpress update—Trends in database growth and links to data analysis tools. Nucleic Acids Res.

[91] Lou S.-K., Ni B., Lo L.-Y., Tsui S.K.-W., Chan T.-F., Leung K.-S. (2011). ABMapper: A suffix array-based tool for multi-location searching and splice-junction mapping. Bioinformatics.

[92] Langmead B., Salzberg S.L. (2012). Fast gapped-read alignment with Bowtie 2. Nat. Meth.

[93] Trapnell C., Roberts A., Goff L., Pertea G., Kim D., Kelley D.R., Pimentel H., Salzberg S.L., Rinn J.L., Pachter L. (2012). Differential gene and transcript expression analysis of RNA-seq experiments with TopHat and Cufflinks. Nat. Protoc.

[94] Wang L., Feng Z., Wang X., Wang X., Zhang X. (2010). DEGseq: An R package for identifying differentially expressed genes from RNA-seq data. Bioinformatics.

[95] Nawrocki E.P., Kolbe D.L., Eddy S.R. (2009). Infernal 1.0: Inference of RNA alignments. Bioinformatics.

[96] Schulz M.H., Zerbino D.R., Vingron M., Birney E. (2012). Oases: Robust *de novo* RNA-seq assembly across the dynamic range of expression levels. Bioinformatics.

[97] Trapnell C., Pachter L., Salzberg S.L. (2009). TopHat: Discovering splice junctions with RNA-Seq. Bioinformatics.

[98] Robertson G., Schein J., Chiu R., Corbett R., Field M., Jackman S.D., Mungall K., Lee S., Okada H.M., Qian J.Q. (2010). *De novo* assembly and analysis of RNA-seq data. Nat. Meth.

[99] Grabherr M.G., Haas B.J., Yassour M., Levin J.Z., Thompson D.A., Amit I., Adiconis X., Fan L., Raychowdhury R., Zeng Q. (2011). Full-length transcriptome assembly from RNA-Seq data without a reference genome. Nat. Biotechnol.

[100] Sekhon R.S., Lin H., Childs K.L., Hansey C.N., Buell C.R., de Leon N., Kaeppler S.M. (2011). Genome-wide atlas of transcription during maize development. Plant J.

[101] Severin A., Woody J., Bolon Y.-T., Joseph B., Diers B., Farmer A., Muehlbauer G., Nelson R., Grant D., Specht J. (2010). RNA-Seq Atlas of *Glycine max*: A guide to the soybean transcriptome. BMC Plant Biol.

[102] Ge Y., Li Y., Zhu Y.-M., Bai X., Lv D.-K., Guo D., Ji W., Cai H. (2010). Global transcriptome profiling of wild soybean (*Glycine soja*) roots under NaHCO_3_ treatment. BMC Plant Biol.

[103] Ma T.-L., Wu W.-H., Wang Y. (2012). Transcriptome analysis of rice root responses to potassium deficiency. BMC Plant Biol.

[104] An D., Yang J., Zhang P. (2012). Transcriptome profiling of low temperature-treated cassava apical shoots showed dynamic responses of tropical plant to cold stress. BMC Genomics.

[105] Zabala G., Zou J., Tuteja J., Gonzalez D., Clough S., Vodkin L. (2006). Transcriptome changes in the phenylpropanoid pathway of *Glycine max* in response to *Pseudomonas syringae* infection. BMC Plant Biol.

[106] Wang C., Chen H., Hao Q., Sha A., Shan Z., Chen L., Zhou R., Zhi H., Zhou X. (2012). Transcript profile of the response of two soybean genotypes to potassium deficiency. PLoS One.

[107] Lenka S.K., Katiyar A., Chinnusamy V., Bansal K.C. (2011). Comparative analysis of drought-responsive transcriptome in Indica rice genotypes with contrasting drought tolerance. Plant Biotechnol. J.

[108] Zhang T., Zhao X., Wang W., Pan Y., Huang L., Liu X., Zong Y., Zhu L., Yang D., Fu B. (2012). Comparative transcriptome profiling of chilling stress responsiveness in two contrasting rice genotypes. PLoS One.

[109] Li Y.C., Meng F.R., Zhang C.Y., Zhang N., Sun M.S., Ren J.P., Niu H.B., Wang X., Yin J. (2012). Comparative analysis of water stress-responsive transcriptomes in drought-susceptible and -tolerant wheat (*Triticum aestivum* L.). J. Plant Biol.

[110] Zahaf O., Blanchet S., de Zelicourt A., Alunni B., Plet J., Laffont C., de Lorenzo L., Imbeaud S., Ichante J.-L., Diet A. (2012). Comparative transcriptomic analysis of salt adaptation in roots of contrasting *Medicago truncatula* genotypes. Mol. Plant.

[111] Puranik S., Jha S., Srivastava P.S., Sreenivasulu N., Prasad M. (2011). Comparative transcriptome analysis of contrasting foxtail millet cultivars in response to short-term salinity stress. J. Plant Physiol.

[112] Delker C., Quint M. (2011). Expression level polymorphisms: Heritable traits shaping natural variation. Trends Plant Sci.

[113] Holloway B., Luck S., Beatty M., Rafalski J.-A., Li B. (2011). Genome-wide expression quantitative trait loci (eQTL) analysis in maize. BMC Genomics.

[114] Wang J., Yu H., Xie W., Xing Y., Yu S., Xu C., Li X., Xiao J., Zhang Q. (2010). A global analysis of QTLs for expression variations in rice shoots at the early seedling stage. Plant J.

[115] Chen X., Hackett C.A., Niks R.E., Hedley P.E., Booth C., Druka A., Marcel T.C., Vels A., Bayer M., Milne I. (2010). An eQTL Analysis of partial resistance to *Puccinia hordei* in barley. PLoS One.

[116] Mann M. (2008). Can proteomics retire the western blot?. J. Proteome Res.

[117] Gygi S.P., Rist B., Gerber S.A., Turecek F., Gelb M.H., Aebersold R. (1999). Quantitative analysis of complex protein mixtures using isotope-coded affinity tags. Nat. Biotechnol.

[118] Ong S.E., Blagoev B., Kratchmarova I., Kristensen D.B., Steen H., Pandey A., Mann M. (2002). Stable isotope labeling by amino acids in cell culture, SILAC, as a simple and accurate approach to expression proteomics. Mol. Cell. Proteomics.

[119] Wiese S., Reidegeld K.A., Meyer H.E., Warscheid B. (2007). Protein labeling by iTRAQ: A new tool for quantitative mass spectrometry in proteome research. Proteomics.

[120] Cox J., Mann M. (2008). MaxQuant enables high peptide identification rates, individualized p.p.b.-range mass accuracies and proteome-wide protein quantification. Nat. Biotechnol.

[121] Old W.M., Meyer-Arendt K., Aveline-Wolf L., Pierce K.G., Mendoza A., Sevinsky J.R., Resing K.A., Ahn N.G. (2005). Comparison of label-free methods for quantifying human proteins by shotgun proteomics. Mol. Cell. Proteomics.

[122] Jorrín J.V., Maldonado A.M., Castillejo M.A. (2007). Plant proteome analysis: A 2006 update. Proteomics.

[123] Jorrin-Novo J.V., Maldonado A.M., Echevarria-Zomeno S., Valledor L., Castillejo M.A., Curto M., Valero J., Sghaier B., Donoso G., Redondo I. (2009). Plant proteomics update (2007–2008): Second-generation proteomic techniques, an appropriate experimental design, and data analysis to fulfill MIAPE standards, increase plant proteome coverage and expand biological knowledge. J. Proteomics.

[124] Kamal A.H.M., Cho K., Kim D.-E., Uozumi N., Chung K.-Y., Lee S.Y., Choi J.-S., Cho S.-W., Shin C.-S., Woo S.H. (2012). Changes in physiology and protein abundance in salt-stressed wheat chloroplasts. Mol. Biol. Rep.

[125] Ahsan N., Nakamura T., Komatsu S. (2012). Differential responses of microsomal proteins and metabolites in two contrasting cadmium (Cd)-accumulating soybean cultivars under Cd stress. Amino Acids.

[126] Wang H., Wang S., Xia Y. (2012). Identification and verification of redox-sensitive proteins in *Arabidopsis thaliana*. Methods Mol. Biol.

[127] Galant A., Koester R.P., Ainsworth E.A., Hicks L.M., Jez J.M. (2012). From climate change to molecular response: Redox proteomics of ozone-induced responses in soybean. New Phytol.

[128] Nakagami H., Sugiyama N., Mochida K., Daudi A., Yoshida Y., Toyoda T., Tomita M., Ishihama Y., Shirasu K. (2010). Large-scale comparative phosphoproteomics identifies conserved phosphorylation sites in plants. Plant Physiol.

[129] Agrawal G.K., Jwa N.-S., Lebrun M.-H., Job D., Rakwal R. (2010). Plant secretome: Unlocking secrets of the secreted proteins. Proteomics.

[130] Alexandersson E., Ashfaq A., Resjö S., Andreasson E. (2013). Plant secretome proteomics. Front. Plant Sci.

[131] Catalá C., Howe K.J., Hucko S., Rose J.K.C., Thannhauser T.W. (2011). Towards characterization of the glycoproteome of tomato (*Solanum lycopersicum*) fruit using Concanavalin A lectin affinity chromatography and LC-MALDI-MS/MS analysis. Proteomics.

[132] Ruiz-May E., Kim S.J., Brandizzi F., Rose J.K.C. (2012). The secreted plant *n*-glycoproteome and associated secretory pathways. Front. Plant Sci.

[133] Pawson T., Nash P. (2000). Protein-protein interactions define specificity in signal transduction. Genes Dev.

[134] Zhang Y., Gao P., Yuan J.S. (2010). Plant protein-protein interaction network and interactome. Curr. Genomics.

[135] Wittig I., Braun H.-P., Schagger H. (2006). Blue native PAGE. Nat. Protoc.

[136] Hue M., Riffle M., Vert J.-P., Noble W. (2010). Large-scale prediction of protein-protein interactions from structures. BMC Bioinforma.

[137] Moal I.H., Agius R., Bates P.A. (2011). Protein-protein binding affinity prediction on a diverse set of structures. Bioinformatics.

[138] Cusick M.E., Yu H., Smolyar A., Venkatesan K., Carvunis A.-R., Simonis N., Rual J.-F., Borick H., Braun P., Dreze M. (2009). Literature-curated protein interaction datasets. Nat. Meth.

[139] Ho C.-L., Wu Y., Shen H.-B., Provart N., Geisler M. (2012). A predicted protein interactome for rice. Rice.

[140] Cui J., Li P., Li G., Xu F., Zhao C., Li Y.H., Yang Z.N., Wang G., Yu Q.B., Li Y.X. (2008). AtPID: *Arabidopsis thaliana* protein interactome database—An integrative platform for plant systems biology. Nucleic Acids Res.

[141] *Arabidopsis* interactome mapping consortium (2011). Evidence for network evolution in an *Arabidopsis* interactome map. Science.

[142] Seo Y.-S., Chern M., Bartley L.E., Han M., Jung K.-H., Lee I., Walia H., Richter T., Xu X., Cao P. (2011). Towards establishment of a rice stress response interactome. PLoS Genet.

[143] Gu H., Zhu P., Jiao Y., Meng Y., Chen M. (2011). PRIN: A predicted rice interactome network. BMC Bioinforma.

[144] Chatr-aryamontri A., Breitkreutz B.-J., Heinicke S., Boucher L., Winter A., Stark C., Nixon J., Ramage L., Kolas N., O’Donnell L. (2013). The BioGRID interaction database: 2013 update. Nucleic Acids Res.

[145] Xenarios I., Salwínski L., Duan X.J., Higney P., Kim S.-M., Eisenberg D. (2002). DIP, the Database of Interacting Proteins: A research tool for studying cellular networks of protein interactions. Nucleic Acids Res.

[146] Mingwei M., Haoyang C., Wen Z., Zhirui Y., Xiao L., Xinjian F., Quansheng F PlaPID: A Database of Protein-Protein Interactions in Plants.

[147] Kerrien S., Aranda B., Breuza L., Bridge A., Broackes-Carter F., Chen C., Duesbury M., Dumousseau M., Feuermann M., Hinz U. (2012). The IntAct molecular interaction database in 2012. Nucleic Acids Res.

[148] Cooper B., Clarke J.D., Budworth P., Kreps J., Hutchison D., Park S., Guimil S., Dunn M., Luginbühl P., Ellero C. (2003). A network of rice genes associated with stress response and seed development. Proc. Natl. Acad. Sci. USA.

[149] Tardif G., Kane N., Adam H., Labrie L., Major G., Gulick P., Sarhan F., Laliberté J.-F. (2007). Interaction network of proteins associated with abiotic stress response and development in wheat. Plant Mol. Biol.

[150] Afzal A.J., Natarajan A., Saini N., Iqbal M.J., Geisler M., El Shemy H.A., Mungur R., Willmitzer L., Lightfoot D.A. (2009). The nematode resistance allele at the *rhg1* locus alters the proteome and primary metabolism of soybean roots. Plant Physiol.

[151] Gendler K., Paulsen T., Napoli C. (2008). ChromDB: The chromatin database. Nucleic Acids Res.

[152] Morison I.M., Ramsay J.P., Spencer H.G. (2005). A census of mammalian imprinting. Trends Genet.

[153] Tsukahara S., Kobayashi A., Kawabe A., Mathieu O., Miura A., Kakutani T. (2009). Bursts of retrotransposition reproduced in *Arabidopsis*. Nature.

[154] Zhang X.Y., Yazaki J., Sundaresan A., Cokus S., Chan S.W.L., Chen H.M., Henderson I.R., Shinn P., Pellegrini M., Jacobsen S.E. (2006). Genome-wide high-resolution mapping and functional analysis of DNA methylation in *Arabidopsis*. Cell.

[155] Zilberman D., Gehring M., Tran R.K., Ballinger T., Henikoff S. (2007). Genome-wide analysis of *Arabidopsis thaliana* DNA methylation uncovers an interdependence between methylation and transcription. Nat. Genet.

[156] Seifert M., Cortijo S., Colome-Tatche M., Johannes F., Roudier F., Colot V. (2012). MeDIP-HMM: Genome-wide identification of distinct DNA methylation states from high-density tiling arrays. Bioinformatics.

[157] Cokus S.J., Feng S.H., Zhang X.Y., Chen Z.G., Merriman B., Haudenschild C.D., Pradhan S., Nelson S.F., Pellegrini M., Jacobsen S.E. (2008). Shotgun bisulphite sequencing of the Arabidopsis genome reveals DNA methylation patterning. Nature.

[158] Bibikova M., Barnes B., Tsan C., Ho V., Klotzle B., Le J.M., Delano D., Zhang L., Schroth G.P., Gunderson K.L. (2011). High density DNA methylation array with single CpG site resolution. Genomics.

[159] Meissner A., Gnirke A., Bell G.W., Ramsahoye B., Lander E.S., Jaenisch R. (2005). Reduced representation bisulfite sequencing for comparative high-resolution DNA methylation analysis. Nucleic Acids Res.

[160] Dowen R.H., Pelizzola M., Schmitz R.J., Lister R., Dowen J.M., Nery J.R., Dixon J.E., Ecker J.R. (2012). Widespread dynamic DNA methylation in response to biotic stress. Proc. Natl. Acad. Sci. USA.

[161] Wang W.S., Pan Y.J., Zhao X.Q., Dwivedi D., Zhu L.H., Ali J., Fu B.Y., Li Z.K. (2011). Drought-induced site-specific DNA methylation and its association with drought tolerance in rice (*Oryza sativa* L.). J. Exp. Bot.

[162] Zhong L., Xu Y.H., Wang J.B. (2009). DNA-methylation changes induced by salt stress in wheat *Triticum aestivum*. Afr. J. Biotechnol.

[163] Calarco J.P., Borges F., Donoghue M.T., van Ex F., Jullien P.E., Lopes T., Gardner R., Berger F., Feijo J.A., Becker J.D. (2012). Reprogramming of DNA methylation in pollen guides epigenetic inheritance via small RNA. Cell.

[164] Holeski L.M., Jander G., Agrawal A.A. (2012). Transgenerational defense induction and epigenetic inheritance in plants. Trends Ecol. Evol.

[165] Kou H.P., Li Y., Song X.X., Ou X.F., Xing S.C., Ma J., von Wettstein D., Liu B. (2011). Heritable alteration in DNA methylation induced by nitrogen-deficiency stress accompanies enhanced tolerance by progenies to the stress in rice (*Oryza sativa* L.). J. Plant Physiol.

[166] Lutsik P., Feuerbach L., Arand J., Lengauer T., Walter J., Bock C. (2011). BiQ Analyzer HT: Locus-specific analysis of DNA methylation by high-throughput bisulfite sequencing. Nucleic Acids Res.

[167] Krueger F., Andrews S.R. (2011). Bismark: A flexible aligner and methylation caller for Bisulfite-Seq applications. Bioinformatics.

[168] Harris E.Y., Ponts N., Le Roch K.G., Lonardi S. (2012). BRAT-BW: Efficient and accurate mapping of bisulfite-treated reads. Bioinformatics.

[169] Hansen K.D., Langmead B., Irizarry R.A. (2012). BSmooth: From whole genome bisulfite sequencing reads to differentially methylated regions. Genome Biol.

[170] Chen P.Y., Cokus S.J., Pellegrini M. (2010). BS Seeker: Precise mapping for bisulfite sequencing. BMC Bioinforma.

[171] Xi Y., Li W. (2009). BSMAP: Whole genome bisulfite sequence MAPping program. BMC Bioinforma.

[172] Su J.Z., Yan H.D., Wei Y.J., Liu H.B., Liu H., Wang F., Lv J., Wu Q., Zhang Y. (2013). CpG_MPs: Identification of CpG methylation patterns of genomic regions from high-throughput bisulfite sequencing data. Nucleic Acids Res.

[173] Benoukraf T., Wongphayak S., Hadi L.H., Wu M., Soong R (2012). GBSA: A comprehensive software for analysing whole genome bisulfite sequencing data. Nucleic Acids Res..

[174] Gruntman E., Qi Y.J., Slotkin R.K., Roeder T., Martienssen R.A., Sachidanandam R. (2008). Kismeth: Analyzer of plant methylation states through bisulfite sequencing. BMC Bioinforma.

[175] Kumaki Y., Oda M., Okano M. (2008). QUMA: Quantification tool for methylation analysis. Nucleic Acids Res.

[176] Xi Y.X., Bock C., Muller F., Sun D.Q., Meissner A., Li W. (2012). RRBSMAP: A fast, accurate and user-friendly alignment tool for reduced representation bisulfite sequencing. Bioinformatics.

[177] Strahl B.D., Allis C.D. (2000). The language of covalent histone modifications. Nature.

[178] Marino-Ramirez L., Levine K.M., Morales M., Zhang S.Y., Moreland R.T., Baxevanis A.D., Landsman D (2011). The histone database: An integrated resource for histones and histone fold-containing proteins. Database-Oxford.

[179] Lee K.K., Workman J.L. (2007). Histone acetyltransferase complexes: One size doesn’t fit all. Nat. Rev. Mol. Cell Biol.

[180] Barski A., Cuddapah S., Cui K.R., Roh T.Y., Schones D.E., Wang Z.B., Wei G., Chepelev I., Zhao K.J. (2007). High-resolution profiling of histone methylations in the human genome. Cell.

[181] Oki M., Aihara H., Ito T, Kundu T., Bittman R., Dasgupta D., Engelhardt H., Flohe L., Herrmann H., Holzenburg A., Nasheuer H.P., Rottem S., Wyss M., Zwickl P. (2007). Role Of Histone Phosphorylation In Chromatin Dynamics And Its Implications in Diseases. Chromatin and Disease.

[182] Shivaswamy S., Iyer V.R. (2007). Genome-wide analysis of chromatin status using tiling microarrays. Methods.

[183] Johnson D.S., Mortazavi A., Myers R.M., Wold B. (2007). Genome-wide mapping of *in vivo* protein-DNA interactions. Science.

[184] Papaefthimiou D., Tsaftaris A.S. (2012). Characterization of a drought inducible trithorax-like H3K4 methyltransferase from barley. Biol. Plant.

[185] Ding B., Bellizzi M.D.R., Ning Y., Meyers B.C., Wang G.-L. (2012). HDT701, a Histone H4 deacetylase, negatively regulates plant innate immunity by modulating histone H4 acetylation of defense-related genes in rice. Plant Cell.

[186] Song Y.G., Ji D.D., Li S., Wang P., Li Q., Xiang F.N. (2012). The dynamic changes of DNA methylation and histone modifications of salt responsive transcription factor genes in soybean. PLoS One.

[187] Zong W., Zhong X., You J., Xiong L. (2013). Genome-wide profiling of histone H3K4-tri-methylation and gene expression in rice under drought stress. Plant Mol. Biol.

[188] Munns R., James R.A., Sirault X.R.R., Furbank R.T., Jones H.G. (2010). New phenotyping methods for screening wheat and barley for beneficial responses to water deficit. J. Exp. Bot.

[189] Golzarian M., Frick R., Rajendran K., Berger B., Roy S., Tester M., Lun D. (2011). Accurate inference of shoot biomass from high-throughput images of cereal plants. Plant Methods.

[190] Paproki A., Sirault X., Berry S., Furbank R., Fripp J. (2012). A novel mesh processing based technique for 3D plant analysis. BMC Plant Biol.

[191] Spielbauer G., Armstrong P., Baier J.W., Allen W.B., Richardson K., Shen B., Settles A.M. (2009). High-throughput near-infrared reflectance spectroscopy for predicting quantitative and qualitative composition phenotypes of individual maize kernels. Cereal Chem.

[192] Baker N.R. (2008). Chlorophyll fluorescence: A probe of photosynthesis *in vivo*. Annu. Rev. Plant Biol.

[193] Condon A.G., Richards R.A., Rebetzke G.J., Farquhar G.D. (2004). Breeding for high water-use efficiency. J. Exp. Bot.

[194] Hargreaves C., Gregory P., Bengough A.G. (2009). Measuring root traits in barley (*Hordeum vulgare* ssp. *vulgare* and ssp. *spontaneum*) seedlings using gel chambers, soil sacs and X-ray microtomography. Plant Soil.

[195] Rajendran K., Tester M., Roy S.J. (2009). Quantifying the three main components of salinity tolerance in cereals. Plant Cell Environ.

[196] Jones H.G., Serraj R., Loveys B.R., Xiong L., Wheaton A., Price A.H. (2009). Thermal infrared imaging of crop canopies for the remote diagnosis and quantification of plant responses to water stress in the field. Funct. Plant Biol.

[197] Moshou D., Bravo C., Oberti R., West J., Bodria L., McCartney A., Ramon H. (2005). Plant disease detection based on data fusion of hyper-spectral and multi-spectral fluorescence imaging using Kohonen maps. Real-Time Imaging.

[198] Genga A., Mattana M., Coraggio I., Locatelli F., Piffanelli P., Consonni R, Shanker A., Venkateswarlu B. (2011). Plant Metabolomics: A Characterisation of Plant Responses to Abiotic Stresses. Abiotic Stress in Plants—Mechanisms and Adaptations.

[199] Kooke R., Keurentjes J.J.B. (2011). Multi-dimensional regulation of metabolic networks shaping plant development and performance. J. Exp. Bot..

[200] Cramer G.R., Urano K., Delrot S., Pezzotti M., Shinozaki K. (2011). Effects of abiotic stress on plants: A systems biology perspective. BMC Plant Biol.

[201] Huang G.-T., Ma S.-L., Bai L.-P., Zhang L., Ma H., Jia P., Liu J., Zhong M., Guo Z.-F. (2012). Signal transduction during cold, salt, and drought stresses in plants. Mol. Biol. Rep.

[202] Liland K.H. (2011). Multivariate methods in metabolomics—From pre-processing to dimension reduction and statistical analysis. Trends Anal. Chem.

[203] Stenlund H., Gorzsas A., Persson P., Sundberg B., Trygg J. (2008). Orthogonal projections to latent structures discriminant analysis modeling on *in situ* FT-IR spectral imaging of liver tissue for identifying sources of variability. Anal. Chem.

[204] Trygg J., Wold S. (2002). Orthogonal projections to latent structures (O-PLS). J. Chemom.

[205] Kopka J., Schauer N., Krueger S., Birkemeyer C., Usadel B., Bergmüller E., Dörmann P., Weckwerth W., Gibon Y., Stitt M. (2005). GMD@CSB.DB: The golm metabolome database. Bioinformatics.

[206] Fahy E., Sud M., Cotter D., Subramaniam S. (2007). LIPID MAPS online tools for lipid research. Nucleic Acids Res.

[207] Cui Q., Lewis I.A., Hegeman A.D., Anderson M.E., Li J., Schulte C.F., Westler W.M., Eghbalnia H.R., Sussman M.R., Markley J.L. (2008). Metabolite identification via the Madison Metabolomics Consortium Database. Nat. Biotechnol.

[208] Brown M., Dunn W.B., Dobson P., Patel Y., Winder C.L., Francis-McIntyre S., Begley P., Carroll K., Broadhurst D., Tseng A. (2009). Mass spectrometry tools and metabolite-specific databases for molecular identification in metabolomics. Analyst.

[209] Horai H., Arita M., Kanaya S., Nihei Y., Ikeda T., Suwa K., Ojima Y., Tanaka K., Tanaka S., Aoshima K. (2010). MassBank: A public repository for sharing mass spectral data for life sciences. J. Mass Spectrom.

[210] Carroll A., Badger M., Harvey Millar A. (2010). The MetabolomeExpress Project: Enabling web-based processing, analysis and transparent dissemination of GC/MS metabolomics datasets. BMC Bioinforma.

[211] Tautenhahn R., Cho K., Uritboonthai W., Zhu Z., Patti G.J., Siuzdak G. (2012). An accelerated workflow for untargeted metabolomics using the METLIN database. Nat. Biotechnol.

[212] Zhang F., Robinette S.L., Bruschweiler-Li L., Brüschweiler R. (2009). Web server suite for complex mixture analysis by covariance NMR. Magn. Reson. Chem.

[213] Biswas A., Mynampati K.C., Umashankar S., Reuben S., Parab G., Rao R., Kannan V.S., Swarup S. (2010). MetDAT: A modular and workflow-based free online pipeline for mass spectrometry data processing, analysis and interpretation. Bioinformatics.

[214] Zhou B., Wang J., Ressom H.W. (2012). MetaboSearch: Tool for mass-based metabolite identification using multiple databases. PLoS One.

[215] Gavaghan C.L., Li J.V., Hadfield S.T., Hole S., Nicholson J.K., Wilson I.D., Howe P.W.A., Stanley P.D., Holmes E. (2011). Application of NMR-based Metabolomics to the Investigation of Salt Stress in Maize (*Zea mays*). Phytochem. Anal.

[216] Widodo Patterson J.H., Newbigin E., Tester M., Bacic A., Roessner U. (2009). Metabolic responses to salt stress of barley (*Hordeum vulgare* L.) cultivars, Sahara and Clipper, which differ in salinity tolerance. J. Exp. Bot..

[217] Wu J., Yu H., Dai H., Mei W., Huang X., Zhu S., Peng M. (2012). Metabolite profiles of rice cultivars containing bacterial blight-resistant genes are distinctive from susceptible rice. Acta Biochim. Biophys. Sin.

[218] Levi A., Paterson A.H., Cakmak I., Saranga Y. (2011). Metabolite and mineral analyses of cotton near-isogenic lines introgressed with QTLs for productivity and drought-related traits. Physiol. Plant.

[219] Semel Y., Schauer N., Roessner U., Zamir D., Fernie A.R. (2007). Metabolite analysis for the comparison of irrigated and non-irrigated field grown tomato of varying genotype. Metabolomics.

[220] Silvente S., Sobolev A.P., Lara M. (2012). Metabolite adjustments in drought tolerant and sensitive soybean genotypes in response to water stress. PLoS One.

[221] Witt S., Galicia L., Lisec J., Cairns J., Tiessen A., Luis Araus J., Palacios-Rojas N., Fernie A.R. (2012). Metabolic and phenotypic responses of greenhouse-grown maize hybrids to experimentally controlled drought stress. Mol. Plant.

[222] Komatsu S., Yamamoto A., Nakamura T., Nouri M.-Z., Nanjo Y., Nishizawa K., Furukawa K. (2011). Comprehensive analysis of mitochondria in roots and hypocotyls of soybean under flooding stress using proteomics and metabolomics techniques. J. Proteome Res.

[223] Cho K., Shibato J., Agrawal G.K., Jung Y.-H., Kubo A., Jwa N.-S., Tamogami S., Satoh K., Kikuchi S., Higashi T. (2008). Integrated transcriptomics, proteomics, and metabolomics analyses to survey ozone responses in the leaves of rice seedling. J. Proteome Res.

[224] Aliferis K.A., Jabaji S. (2012). FT-ICR/MS and GC-EI/MS metabolomics networking unravels global potato sprout’s responses to *Rhizoctonia solani* infection. PLoS One.

[225] Figueiredo A., Fortes A.M., Ferreira S., Sebastiana M., Choi Y.H., Sousa L., Acioli-Santos B., Pessoa F., Verpoorte R., Pais M.S. (2008). Transcriptional and metabolic profiling of grape (*Vitis vinifera* L.) leaves unravel possible innate resistance against pathogenic fungi. J. Exp. Bot.

[226] Hong Y.-S., Martinez A., Liger-Belair G., Jeandet P., Nuzillard J.-M., Cilindre C. (2012). Metabolomics reveals simultaneous influences of plant defence system and fungal growth in *Botrytis cinerea*-infected *Vitis vinifera* cv. Chardonnay berries. J. Exp. Bot.

[227] Cevallos-Cevallos J.M., Futch D.B., Shilts T., Folimonova S.Y., Reyes-De-Corcuera J.I. (2012). GC-MS metabolomic differentiation of selected citrus varieties with different sensitivity to citrus huanglongbing. Plant Physiol. Biochem.

[228] Ali K., Maltese F., Figueiredo A., Rex M., Fortes A.M., Zyprian E., Pais M.S., Verpoorte R., Choi Y.H. (2012). Alterations in grapevine leaf metabolism upon inoculation with *Plasmopara viticola* in different time-points. Plant Sci.

[229] Fumagalli E., Baldoni E., Abbruscato P., Piffanelli P., Genga A., Lamanna R., Consonni R. (2009). NMR techniques coupled with multivariate statistical analysis: Tools to analyse *Oryza sativa* metabolic content under stress conditions. J. Agron. Crop Sci.

[230] Rose M.T., Rose T.J., Pariasca-Tanaka J., Yoshihashi T., Neuweger H., Goesmann A., Frei M., Wissuwa M. (2012). Root metabolic response of rice (*Oryza sativa* L.) genotypes with contrasting tolerance to zinc deficiency and bicarbonate excess. Planta.

[231] Shulaev V. (2006). Metabolomics technology and bioinformatics. Brief. Bioinforma.

[232] Wu W., Zhang Q., Zhu Y., Lam H.-M., Cai Z., Guo D. (2008). Comparative metabolic profiling reveals secondary metabolites correlated with soybean salt tolerance. J. Agric. Food Chem.

[233] Johnson H.E., Broadhurst D., Goodacre R., Smith A.R. (2003). Metabolic fingerprinting of salt-stressed tomatoes. Phytochemistry.

[234] Liu L., Li Y.H., Li S.L., Hu N., He Y.M., Pong R., Lin D.N., Lu L.H., Law M (2012). Comparison of next-generation sequencing systems. J. Biomed. Biotechnol..

[235] Kao H.-L., Gunsalus K.C. (2002). Browsing Multidimensional Molecular Networks with the Generic Network Browser (N-Browse). Current Protocols in Bioinformatics.

[236] Smoot M.E., Ono K., Ruscheinski J., Wang P.-L., Ideker T. (2011). Cytoscape 2.8: New features for data integration and network visualization. Bioinformatics.

[237] Katari M.S., Nowicki S.D., Aceituno F.F., Nero D., Kelfer J., Thompson L.P., Cabello J.M., Davidson R.S., Goldberg A.P., Shasha D.E. (2010). VirtualPlant: A software platform to support systems biology research. Plant Physiol.

[238] Jami S.K., Clark G.B., Ayele B.T., Ashe P., Kirti P.B. (2012). Genome-wide comparative analysis of annexin superfamily in plants. PLoS One.

[239] Wan H., Yuan W., Bo K., Shen J., Pang X., Chen J. (2013). Genome-wide analysis of NBS-encoding disease resistance genes in *Cucumis sativus* and phylogenetic study of NBS-encoding genes in Cucurbitaceae crops. BMC Genomics.

[240] Hu L., Liu S. (2011). Genome-wide identification and phylogenetic analysis of the ERF gene family in cucumbers. Genet. Mol. Biol.

[241] Li Q., Zhang C., Li J., Wang L., Ren Z. (2012). Genome-wide identification and characterization of R2R3MYB family in *Cucumis sativus*. PLoS One.

[242] Ling J., Jiang W., Zhang Y., Yu H., Mao Z., Gu X., Huang S., Xie B. (2011). Genome-wide analysis of *WRKY* gene family in *Cucumis sativus*. BMC Genomics.

[243] Kang Y., Kim K., Shim S., Yoon M., Sun S., Kim M., Van K., Lee S.-H. (2012). Genome-wide mapping of NBS-LRR genes and their association with disease resistance in soybean. BMC Plant Biol.

[244] Du H., Yang S.-S., Liang Z., Feng B.-R., Liu L., Huang Y.-B., Tang Y.-X. (2012). Genome-wide analysis of the MYB transcription factor superfamily in soybean. BMC Plant Biol.

[245] Dung Tien L., Nishiyama R., Watanabe Y., Mochida K., Yamaguchi-Shinozaki K., Shinozaki K., Lam-Son Phan T. (2011). Genome-wide survey and expression analysis of the plant-specific NAC transcription factor family in soybean during development and dehydration stress. DNA Res.

[246] Osorio M.B., Buecker-Neto L., Castilhos G., Turchetto-Zolet A.C., Wiebke-Strohm B., Bodanese-Zanettini M.H., Margis-Pinheiro M. (2012). Identification and *in silico* characterization of soybean trihelix-GT and bHLH transcription factors involved in stress responses. Genet. Mol. Biol.

[247] Tran L.-S.P., Quach T.N., Guttikonda S.K., Aldrich D.L., Kumar R., Neelakandan A., Valliyodan B., Nguyen H.T. (2009). Molecular characterization of stress-inducible *GmNAC* genes in soybean. Mol. Genet. Genomics.

[248] Zhou Q.-Y., Tian A.-G., Zou H.-F., Xie Z.-M., Lei G., Huang J., Wang C.-M., Wang H.-W., Zhang J.-S., Chen S.-Y. (2008). Soybean WRKY-type transcription factor genes, *GmWRKY13*, *GmWRKY21*, and *GmWRKY54*, confer differential tolerance to abiotic stresses in transgenic *Arabidopsis* plants. Plant Biotechnol. J.

[249] Liang D., Xia H., Wu S., Ma F. (2012). Genome-wide identification and expression profiling of dehydrin gene family in *Malus domestica*. Mol. Biol. Rep.

[250] Zhao T., Liang D., Wang P., Liu J., Ma F. (2012). Genome-wide analysis and expression profiling of the DREB transcription factor gene family in *Malus* under abiotic stress. Mol. Genet. Genomics.

[251] Agalou A., Purwantomo S., Oevernaes E., Johannesson H., Zhu X., Estiati A., de Kam R.J., Engstroem P., Slamet-Loedin I.H., Zhu Z. (2008). A genome-wide survey of HD-Zip genes in rice and analysis of drought-responsive family members. Plant Mol. Biol.

[252] Agarwal P., Arora R., Ray S., Singh A.K., Singh V.P., Takatsuji H., Kapoor S., Tyagi A.K. (2007). Genome-wide identification of C2H2 zinc-finger gene family in rice and their phylogeny and expression analysis. Plant Mol. Biol.

[253] Amrutha R.N., Sekhar P.N., Varshney R.K., Kishor P.B.K. (2007). Genome-wide analysis and identification of genes related to potassium transporter families in rice (*Oryza sativa* L.). Plant Sci.

[254] Chen R., Jiang Y., Dong J., Zhang X., Xiao H., Xu Z., Gao X. (2012). Genome-wide analysis and environmental response profiling of SOT family genes in rice (*Oryza sativa*). Genes Genomics.

[255] Ding X., Hou X., Xie K., Xiong L. (2009). Genome-wide identification of BURP domain-containing genes in rice reveals a gene family with diverse structures and responses to abiotic stresses. Planta.

[256] Gollan P.J., Bhave M. (2010). Genome-wide analysis of genes encoding FK506-binding proteins in rice. Plant Mol. Biol.

[257] Huang J., Zhao X., Yu H., Ouyang Y., Wang L., Zhang Q. (2009). The ankyrin repeat gene family in rice: Genome-wide identification, classification and expression profiling. Plant Mol. Biol.

[258] Jain M., Tyagi A.K., Khurana J.P. (2008). Genome-wide identification, classification, evolutionary expansion and expression analyses of homeobox genes in rice. FEBS J.

[259] Jiang S.-Y., Ramamoorthy R., Bhalla R., Luan H.-F., Venkatesh P.N., Cai M., Ramachandran S. (2008). Genome-wide survey of the RIP domain family in *Oryza sativa* and their expression profiles under various abiotic and biotic stresses. Plant Mol. Biol.

[260] Nuruzzaman M., Manimekalai R., Sharoni A.M., Satoh K., Kondoh H., Ooka H., Kikuchi S. (2010). Genome-wide analysis of NAC transcription factor family in rice. Gene.

[261] Nuruzzaman M., Sharoni A.M., Satoh K., Al-Shammari T., Shimizu T., Sasaya T., Omura T., Kikuchi S. (2012). The thioredoxin gene family in rice: Genome-wide identification and expression profiling under different biotic and abiotic treatments. Biochem. Biophys. Res. Commun.

[262] Ouyang Y., Chen J., Xie W., Wang L., Zhang Q. (2009). Comprehensive sequence and expression profile analysis of Hsp20 gene family in rice. Plant. Mol. Biol.

[263] Vij S., Giri J., Dansana P.K., Kapoor S., Tyagi A.K. (2008). The receptor-like cytoplasmic kinase (OsRLCK) gene family in rice: Organization, phylogenetic relationship, and expression during development and stress. Mol. Plant.

[264] Wang D., Guo Y., Wu C., Yang G., Li Y., Zheng C. (2008). Genome-wide analysis of CCCH zinc finger family in *Arabidopsis* and rice. BMC Genomics.

[265] Zhao H., Ma H., Yu L., Wang X., Zhao J. (2012). Genome-wide survey and expression analysis of amino acid transporter gene family in rice (*Oryza sativa* L.). PLoS One.

[266] Wu J., Peng Z., Liu S., He Y., Cheng L., Kong F., Wang J., Lu G. (2012). Genome-wide analysis of Aux/IAA gene family in *Solanaceae* species using tomato as a model. Mol. Genet. Genomics.

[267] Bai M., Yang G.-S., Chen W.-T., Mao Z.-C., Kang H.-X., Chen G.-H., Yang Y.-H., Xie B.-Y. (2012). Genome-wide identification of Dicer-like, Argonaute and RNA-dependent RNA polymerase gene families and their expression analyses in response to viral infection and abiotic stresses in *Solanum lycopersicum*. Gene.

[268] Huang S., Gao Y., Liu J., Peng X., Niu X., Fei Z., Cao S., Liu Y. (2012). Genome-wide analysis of WRKY transcription factors in *Solanum lycopersicum*. Mol. Genet. Genomics.

[269] Kong F., Wang J., Cheng L., Liu S., Wu J., Peng Z., Lu G. (2012). Genome-wide analysis of the mitogen-activated protein kinase gene family in *Solanum lycopersicum*. Gene.

[270] Gan D., Jiang H., Zhang J., Zhao Y., Zhu S., Cheng B. (2011). Genome-wide analysis of BURP domain-containing genes in Maize and Sorghum. Mol. Biol. Rep.

[271] Vannozzi A., Dry I.B., Fasoli M., Zenoni S., Lucchin M. (2012). Genome-wide analysis of the grapevine stilbene synthase multigenic family: Genomic organization and expression profiles upon biotic and abiotic stresses. BMC Plant Biol.

[272] Zhuang J., Peng R.-H., Cheng Z.-M., Zhang J., Cai B., Zhang Z., Gao F., Zhu B., Fu X.-Y., Jin X.-F. (2009). Genome-wide analysis of the putative AP2/ERF family genes in *Vitis vinifera*. Sci. Hortic.

[273] Cheng Y., Li X., Jiang H., Ma W., Miao W., Yamada T., Zhang M. (2012). Systematic analysis and comparison of nucleotide-binding site disease resistance genes in maize. FEBS J.

[274] Gomez-Anduro G., Adriana Ceniceros-Ojeda E., Edith Casados-Vazquez L., Bencivenni C., Sierra-Beltran A., Murillo-Amador B., Tiessen A. (2011). Genome-wide analysis of the beta-glucosidase gene family in maize (*Zea mays* L. var B73). Plant Mol. Biol.

[275] Lin Y.-X., Jiang H.-Y., Chu Z.-X., Tang X.-L., Zhu S.-W., Cheng B.-J. (2011). Genome-wide identification, classification and analysis of heat shock transcription factor family in maize. BMC Genomics.

[276] Peng X., Zhao Y., Cao J., Zhang W., Jiang H., Li X., Ma Q., Zhu S., Cheng B. (2012). CCCH-type zinc finger family in Maize: Genome-wide identification, classification and expression profiling under abscisic acid and drought treatments. PLoS One.

[277] Wang W.W., Ma Q., Xiang Y., Zhu S.W., Cheng B.J. (2012). Genome-wide analysis of immunophilin *FKBP* genes and expression patterns in *Zea may*s. Genet. Mol. Res.

[278] Wei K.-F., Chen J., Chen Y.-F., Wu L.-J., Xie D.-X. (2012). Molecular phylogenetic and expression analysis of the complete WRKY transcription factor family in Maize. DNA Res.

[279] Zhang Z., Zhang J., Chen Y., Li R., Wang H., Wei J. (2012). Genome-wide analysis and identification of HAK potassium transporter gene family in maize (*Zea mays* L.). Mol. Biol. Rep.

[280] Zhou M.-L., Zhang Q., Zhou M., Sun Z.-M., Zhu X.-M., Shao J.-R., Tang Y.-X., Wu Y.-M. (2012). Genome-wide identification of genes involved in raffinose metabolism in Maize. Glycobiology.

[281] Wendelboe-Nelson C., Morris P.C. (2012). Proteins linked to drought tolerance revealed by DIGE analysis of drought resistant and susceptible barley varieties. Proteomics.

[282] Fatehi F., Hosseinzadeh A., Alizadeh H., Brimavandi T., Struik P.C. (2012). The proteome response of salt-resistant and salt-sensitive barley genotypes to long-term salinity stress. Mol. Biol. Rep.

[283] Cheng Z.Y., Woody O.Z., McConkey B.J., Glick B.R. (2012). Combined effects of the plant growth-promoting bacterium *Pseudomonas putida* UW4 and salinity stress on the *Brassica napus* proteome. Appl. Soil Ecol.

[284] Louarn S., Nawrocki A., Edelenbos M., Jensen D.F., Jensen O.N., Collinge D.B., Jensen B. (2012). The influence of the fungal pathogen *Mycocentrospora acerina* on the proteome and polyacetylenes and 6-methoxymellein in organic and conventionally cultivated carrots (*Daucus carota*) during post harvest storage. J. Proteomics.

[285] Deeba F., Pandey A.K., Ranjan S., Mishra A., Singh R., Sharma Y.K., Shirke P.A., Pandey V. (2012). Physiological and proteomic responses of cotton (*Gossypium herbaceum* L.) to drought stress. Plant Physiol. Biochnol.

[286] Zheng M., Wang Y.H., Liu K., Shu H.M., Zhou Z.G. (2012). Protein expression changes during cotton fiber elongation in response to low temperature stress. J. Plant Physiol.

[287] Wang Y.H., Zheng M., Gao X.B., Zhou Z.G. (2012). Protein differential expression in the elongating cotton (*Gossypium hirsutum* L.) fiber under nitrogen stress. Sci. China Life Sci.

[288] Li J., Sun J., Yang Y.J., Guo S.R., Glick B.R. (2012). Identification of hypoxic-responsive proteins in cucumber roots using a proteomic approach. Plant Physiol. Biochnol.

[289] Palmieri M.C., Perazzolli M., Matafora V., Moretto M., Bachi A., Pertot I. (2012). Proteomic analysis of grapevine resistance induced by *Trichoderma harzianum* T39 reveals specific defence pathways activated against downy mildew. J. Exp. Bot.

[290] Wang Z., Zhao F.X., Zhao X., Ge H., Chai L.J., Chen S.W., Perl A., Ma H.Q. (2012). Proteomic analysis of berry-sizing effect of GA3 on seedless *Vitis vinifera* L. Proteomics.

[291] Minas I.S., Tanou G., Belghazi M., Job D., Manganaris G.A., Molassiotis A., Vasilakakis M. (2012). Physiological and proteomic approaches to address the active role of ozone in kiwifruit post-harvest ripening. J. Exp. Bot.

[292] Huang H., Moller I.M., Song S.Q. (2012). Proteomics of desiccation tolerance during development and germination of maize embryos. J. Proteomics.

[293] Benesova M., Hola D., Fischer L., Jedelsky P.L., Hnilicka F., Wilhelmova N., Rothova O., Kocova M., Prochazkova D., Honnerova J. (2012). The physiology and proteomics of drought tolerance in Maize: Early stomatal closure as a cause of lower tolerance to short-term dehydration?. PLoS One.

[294] Fristedt R., Wasilewska W., Romanowska E., Vener A.V. (2012). Differential phosphorylation of thylakoid proteins in mesophyll and bundle sheath chloroplasts from maize plants grown under low or high light. Proteomics.

[295] Muneer S., Kim T.H., Qureshi M.I. (2012). Fe modulates Cd-induced oxidative stress and the expression of stress responsive proteins in the nodules of *Vigna radiata*. Plant Growth Regul.

[296] Rodrigues S.P., Ventura J.A., Aguilar C., Nakayasu E.S., Choi H., Sobreira T.J.P., Nohara L.L., Wermelinger L.S., Almeida I.C., Zingali R.B. (2012). Label-free quantitative proteomics reveals differentially regulated proteins in the latex of sticky diseased *Carica papaya* L. plants. J. Proteomics.

[297] Mohammadi P.P., Moieni A., Komatsu S. (2012). Comparative proteome analysis of drought-sensitive and drought-tolerant rapeseed roots and their hybrid F1 line under drought stress. Amino Acids.

[298] Zhu M.M., Dai S.J., Zhu N., Booy A., Simons B., Yi S., Chen S.X. (2012). Methyl jasmonate responsive proteins in *Brassica napus* guard cells revealed by iTRAQ-based quantitative proteomics. J. Proteome Res.

[299] Chen J.H., Tian L., Xu H.F., Tian D.G., Luo Y.M., Ren C.M., Yang L.M., Shi J.S. (2012). Cold-induced changes of protein and phosphoprotein expression patterns from rice roots as revealed by multiplex proteomic analysis. Plant Omics.

[300] Ji K.X., Wang Y.Y., Sun W.N., Lou Q.J., Mei H.W., Shen S.H., Chen H. (2012). Drought-responsive mechanisms in rice genotypes with contrasting drought tolerance during reproductive stage. J. Plant Physiol.

[301] Mirzaei M., Soltani N., Sarhadi E., Pascovici D., Keighley T., Salekdeh G.H., Haynes P.A., Atwell B.J. (2012). Shotgun proteomic analysis of long-distance drought signaling in rice roots. J. Proteome Res.

[302] Koga H., Dohi K., Nishiuchi T., Kato T., Takahara H., Mori M., Komatsu S. (2012). Proteomic analysis of susceptible rice plants expressing the whole plant-specific resistance against *Magnaporthe oryzae*: Involvement of a thaumatin-like protein. Physiol. Mol. Plant P.

[303] Li Y.F., Zhang Z.H., Nie Y.F., Zhang L.H., Wang Z.Z. (2012). Proteomic analysis of salicylic acid-induced resistance to *Magnaporthe oryzae* in susceptible and resistant rice. Proteomics.

[304] Hakeem K.R., Chandna R., Ahmad A., Qureshi M.I., Iqbal M. (2012). Proteomic analysis for low and high nitrogen-responsive proteins in the leaves of rice genotypes grown at three nitrogen levels. Appl. Biochem. Biotechnol.

[305] Sawada H., Komatsu S., Nanjo Y., Khan N.A., Kohno Y. (2012). Proteomic analysis of rice response involved in reduction of grain yield under elevated ozone stress. Environ. Exp. Bot.

[306] Wang Y.D., Wang X., Wong Y.S. (2012). Proteomics analysis reveals multiple regulatory mechanisms in response to selenium in rice. J. Proteomics.

[307] Li D.X., Wang L.J., Teng S.L., Zhang G.G., Guo L.J., Mao Q., Wang W., Li M., Chen L. (2012). Proteomics analysis of rice proteins up-regulated in response to bacterial leaf streak disease. J. Plant Biol.

[308] Ngara R., Ndimba R., Borch-Jensen J., Jensen O.N., Ndimba B. (2012). Identification and profiling of salinity stress-responsive proteins in *Sorghum bicolor* seedlings. J. Proteomics.

[309] Hossain Z., Hajika M., Komatsu S. (2012). Comparative proteome analysis of high and low cadmium accumulating soybeans under cadmium stress. Amino Acids.

[310] Mohammadi P.P., Moieni A., Hiraga S., Komatsu S. (2012). Organ-specific proteomic analysis of drought-stressed soybean seedlings. J. Proteomics.

[311] Salavati A., Khatoon A., Nanjo Y., Komatsu S. (2012). Analysis of proteomic changes in roots of soybean seedlings during recovery after flooding. J. Proteomics.

[312] Yanagawa Y., Komatsu S. (2012). Ubiquitin/proteasome-mediated proteolysis is involved in the response to flooding stress in soybean roots, independent of oxygen limitation. Plant Sci.

[313] Khatoon A., Rehman S., Salavati A., Komatsu S. (2012). A comparative proteomics analysis in roots of soybean to compatible symbiotic bacteria under flooding stress. Amino Acids.

[314] Wang L.Q., Ma H., Song L.R., Shu Y.J., Gu W.H. (2012). Comparative proteomics analysis reveals the mechanism of pre-harvest seed deterioration of soybean under high temperature and humidity stress. J. Proteomics.

[315] Wang Y., Yuan X.Z., Hu H., Liu Y., Sun W.H., Shan Z.H., Zhou X.A. (2012). Proteomic analysis of differentially expressed proteins in resistant soybean leaves after *Phakopsora pachyrhizi* infection. J. Phytopathol.

[316] Ma H., Song L., Shu Y., Wang S., Niu J., Wang Z., Yu T., Gu W., Ma H. (2012). Comparative proteomic analysis of seedling leaves of different salt tolerant soybean genotypes. J. Proteomics.

[317] Khatoon A., Rehman S., Hiraga S., Makino T., Komatsu S. (2012). Organ-specific proteomics analysis for identification of response mechanism in soybean seedlings under flooding stress. J. Proteomics.

[318] Nanjo Y., Skultety L., Uvackova L., Kubicova K., Hajduch M., Komatsu S. (2012). Mass spectrometry-based analysis of proteomic changes in the root tips of flooded soybean seedlings. J. Proteome Res.

[319] Khatoon A., Rehman S., Oh M.W., Woo S.H., Komatsu S. (2012). Analysis of response mechanism in soybean under low oxygen and flooding stresses using gel-base proteomics technique. Mol. Biol. Rep.

[320] Koehler G., Wilson R.C., Goodpaster J.V., Sonsteby A., Lai X., Witzmann F.A., You J.S., Rohloff J., Randall S.K., Alsheikh M. (2012). Proteomic study of low-temperature responses in strawberry cultivars (*Fragaria x ananassa*) that differ in cold tolerance. Plant Physiol.

[321] Fang X.P., Chen W.Y., Xin Y., Zhang H.M., Yan C.Q., Yu H., Liu H., Xiao W.F., Wang S.Z., Zheng G.Z. (2012). Proteomic analysis of strawberry leaves infected with *Colletotrichum fragariae*. J. Proteomics.

[322] Zhou G., Yang L.T., Li Y.R., Zou C.L., Huang L.P., Qiu L.H., Huang X., Srivastava M.K. (2012). Proteomic analysis of osmotic stress-responsive proteins in sugarcane leaves. Plant Mol. Biol. Rep.

[323] Shah P., Powell A.L.T., Orlando R., Bergmann C., Gutierrez-Sanchez G. (2012). Proteomic analysis of ripening tomato fruit infected by *Botrytis cinerea*. J. Proteome Res.

[324] Ge P., Ma C., Wang S., Gao L., Li X., Guo G., Ma W., Yan Y. (2012). Comparative proteomic analysis of grain development in two spring wheat varieties under drought stress. Anal. Bioanal. Chem.

[325] Kang G.Z., Li G.Z., Xu W., Peng X.Q., Han Q.X., Zhu Y.J., Guo T.C. (2012). Proteomics reveals the effects of salicylic acid on growth and tolerance to subsequent drought stress in wheat. J. Proteome Res.

[326] Vitamvas P., Prasil I.T., Kosova K., Planchon S., Renaut J. (2012). Analysis of proteome and frost tolerance in chromosome 5A and 5B reciprocal substitution lines between two winter wheats during long-term cold acclimation. Proteomics.

[327] Gunnaiah R., Kushalappa A.C., Duggavathi R., Fox S., Somers D.J. (2012). Integrated metabolo-proteomic approach to decipher the mechanisms by which wheat QTL (Fhb1) contributes to resistance against *Fusarium graminearum*. PLoS One.

[328] Ravalason H., Grisel S., Chevret D., Favel A., Berrin J.G., Sigoillot J.C., Herpoel-Gimbert I. (2012). *Fusarium verticillioides* secretome as a source of auxiliary enzymes to enhance saccharification of wheat straw. Bioresour. Technol.

[329] Kang G.Z., Li G.Z., Zheng B.B., Han Q.X., Wang C.Y., Zhu Y.J., Guo T.C. (2012). Proteomic analysis on salicylic acid-induced salt tolerance in common wheat seedlings (*Triticum aestivum* L.). Biochim. Biophys. Acta.

