# Supplementary material for: Silicon Era of Carbon-Based Life: Application of Genomics and Bioinformatics in Crop Stress Research
Source: Int J Mol Sci. 2013 May 29;14(6):11444–83. doi: 10.3390/ijms140611444 (PMC3709742; doi:10.3390/ijms140611444)
Supplement: Supplementary file 1 [file ijms-14-11444-s001.pdf]

Supplementary Information

Table S1. Crops with reduced genetic diversity proven by whole genome sequencing studies.

Crop	Materials	Methods	Genetic diversity indicators	Values	Reference
**Barley**	Barley cultivar (cv.) Morex (as *de novo* reference) 4 Barley cultivars ("Bowman", "Barke, Igri" and "Haruna Nijo") 1 *Hordeum vulgare* ssp. *spontaneum* (wild progenitor of cultivated barley)	Mapping sequence reads of 4 barley cultivars and *H. spontaneum* accession to the Morex reference genome to identify single nucleotide variation (SNV)	Single nucleotide variation compared with the reference	Bowman: 3,112,520<br>Barke: 3,651,330<br>Haruna Nijao: 948,722<br>Igri: 3,257,946<br>*H. spontaneum*: 6,191,10	[19]
**Maize**	35 improved maize lines 23 traditional landraces 17 wild relatives (14 *parviglumis*; 2 *Zea mays* ssp. *Mexicana*, 1 *Tripsacum dactylodies* var. *meridionale*)	Mapping of re-sequencing read to the maize reference genome (release 4a.53)	LOWESS curves of nucleotide diversity (π)		[42]
**Rice**	40 cultivated rice accessions (*Oryza sativa*) 10 wild progenitors (*Oryza rufipogon* and *Oryza nivara*)	Mapping of re-sequencing reads to IRGSP v4 rice genome	π per kb θ_w per kb	Cultivated rice: 5.4<br>Wilde rice: 7.7<br>Cultivated rice: 6.6<br>Wilde rice: 11.5	[41]
**Soybean**	17 wild soybeans 14 cultivated soybeans	Mapping re-sequencing reads of the 31 accessions tt the Williams 82 reference genome	Whole genome θ_π values	Cultivated: 1.89×10^{-3}<br>Wild: 2.97×10^{-3}	[40]

Table S2. Recent genome-wide identification of gene families.

Species	Gene family	No. of gene	Search strategy	Tools/databases	Reference
Viridiplantae	Annexin Superfamily	149 *	Keyword	SUPERFAMILY database; Plaza database; Phytozome database	[238]
Cucumis sativus	NBS-encoding genes	57	Pattern	TBLASTN; BLASTN; FGENESH; GENSCAN	[239]
	ERF	103	Pattern	TBLASTN; Pfam; SMART	[240]
	R2R3MYB family	55	Pattern	BLASTP; Pfam; SMART	[241]
	WRKY	55	Pattern	BLASTP; HMMER; Pfam	[242]
Glycine max	NB-LRR	319	Pfam ID		[243]
	MYB	244	Pattern	BLASTN; BLASTP; SMART;	[244]
	NAC	152	Other	SoyDB; PlantTFDB; TMHHM	[245]
	trihelix-GT	63	Pattern	BLASTP; SMART; MEME; TBLASTN	[246]
	bHLH	45	Pattern	BLASTP; SMART; MEME; TBLASTN	[246]
	NAC	31	Pattern	TBLASTN	[247]
	WRKY	58	Pattern	BLAST	[248]
Malus domestica	Dehydrin	12	Pattern	Pfam; HMMER	[249]
	DREB	68	Pattern	BLASTP; Pfam; SMART	[250]
Oryza sativa	HD-Zip	30	Pattern	BLAST; GENSCAN	[251]
	C2H2 zinc finger	189	Keyword and Pattern	HMMER; PROSITE; INTERPRO	[252]
	HAK Potassium transporter gene	26	Pattern	BLAST; GENSCAN; GenomeScan; FGENESH; GeneMark.hmm; GrailEXP	[253]
	SOT family	35	Pattern	BLAST; SMART; Pfam	[254]
	BURP domain-containing genes	17	Pattern	BLAST; Pfam; SMART	[255]
	FKBP	29	Keyword and Pattern	TBLASTN	[256]
	Ankyrin repeat containing proteins	158	Pattern	BLASTP; TBLASTN; Pfam; hmmemit	[257]
Oryza sativa	Homeobox	107	Pattern	Pfam; SMART; TIGR; BLAST	[258]
	Ribosome-inactivating proteins	31	Keyword and Pattern	TBLASTN; BLASTP; Pfam	[259]
	NAC	151	Keyword and Pattern	BLASTP; TBLASTN; Pfam; SMART	[260]
	Thioredoxin gene	61	Other	HomoloGene	[261]
	Hsp20	39	Pattern	Pfam; BlastP; SMART	[262]
	AP2/EREBP	163	Keyword and Pattern	BLASTP; TBLASTN; SMART; Pfam	[87]
	Receptor-like cytoplasmic kinase	379	Pattern	HMMER; SMART; InterProScan; TMMH2; KOME	[263]
	CCCH-type zinc finger	67	Pattern	SMART; Pfam; BLASTP; TBLASTN	[264]
	Amino acid transporter	85	Keyword and Pattern	BLASTP; Pfam; InterProScan	[265]

Table S2. *Cont.*

Species	Gene family	No. of gene	Search strategy	Tools/databases	Reference
Solanaceae sp.	Aux/IAA gene	26 + 27	Keyword and Pattern	TBLASTN; DNASTAR; FGENESH	[266]
Solanum lycopersicum	Dicer-like	7	Pattern	Pfam; HMMER; BLAST	[267]
	Argonaute	15	Pattern	Pfam; HMMER; BLAST	[267]
	RNA-dependent RNA polyerase	6	Pattern	Pfam; HMMER; BLAST	[267]
	WRKY	81	Pattern	Pfam; HMMER; SMART	[268]
	MAP Kinase	16	Pattern	SOL Genomics Network; tomato plant GDB database	[269]
Sorghum vulgare	BURP domain-containing genes	11	Pattern	Pfam; SMART: BLASTP	[270]
Vitis vinifera	Stilbene synthase multigenic family	48	Pattern	BLAST; GAZE; JIGSAW; Genoscope	[271]
	AP2/ERF	132	Pattern	BLASTP; PSI-BLAST	[272]
Zea mays	NBS-encoding gene	109	Pattern	Pfam; DNATOOLS; SMART	[273]
	BURP domain-containing genes	15	Pattern	Pfam; SMART: BLASTP	[270]
	beta-glucosidase	26	Pattern	BLAST	[274]
	Heat shock transcription factor	25	Pattern	BLASTP; Pfam; SMART	[275]
	CCCH-type zinc finger	68	Pattern	BLASTP; DNATOOLS software; Pfam; SMART	[276]
	immunophilin FKBP	30	Keyword and Pattern	BLASTP; Pfam	[277]
	WRKY	119	Pattern	BLASTP	[278]
	HAK potassium transporter gene	27	Pattern	BLASTP	[279]
	Galactinol synthase	53	Pattern	MaizeGDB; Maize Sequence Search; Pfam	[280]
	RS and stachyose synthase	10	Pattern	MaizeGDB; Maize Sequence Search; Pfam	[280]
	alpha-galactosidase	5	Pattern	MaizeGDB; Maize Sequence Search; Pfam	[280]
	beta-fructofuranosidase	11	Pattern	MaizeGDB; Maize Sequence Search; Pfam	[280]

* Total number of genes identified from multiple Viridiplantae species.

Table S3. Proteomics studies related to crop stresses reported in 2012.

Species	Stress	Analysis methods	Reference
Barley	Drought	2D DIGE + MALDI-TOF MS	[281]
	Salt	2D PAGE + MALDI-TOF-TOF MS	[282]
Canola	*Pseudomonas putida* + Salt	2D DIGE + Q-TOF, LC-MS/MS	[283]
Carrot	*Mycocentrospora acerina*	2D PAGE + MALDI-TOF-TOF MS	[284]
Cotton	Drought	2D PAGE + MALDI-TOF-TOF MS	[285]
	Low temperature	2D PAGE + MALDI-TOF MS, MALDI-TOF-TOF MS	[286]
	Nitrogen stress	2D PAGE + MALDI-TOF MS, MALDI-TOF-TOF MS	[287]
Cucumber	Hypoxic stress	2D PAGE + MALDI-TOF MS, MALDI-TOF-TOF MS	[288]
Grapevine	Downy mildew	Eight-plex + LC-MS/MS	[289]
	GA3	2D PAGE + MALDI-TOF MS, MALDI-TOF-TOF MS	[290]
Kiwifruit	Ozone	SDS PAGE + LC-MS/MS	[291]
Maize	Desiccation	2D PAGE + MALDI-TOF-TOF MS	[292]
	Drought	iTRAQ + LC-MALDI	[293]
	Low light	SDS PAGE (ProQ Diamond staining for phosphoprotein or Western blot with antibody against phsophothreonine) LC-MS/MS, thylakoid proteins	[294]
Mungbean	Cd-induced oxidative stress	2D PAGE + LC-MS/MS	[295]
Papaya	Papaya meleira virus	SDS PAGE + LC-MS/MS	[296]
Rapeseed	Drought	2D PAGE + LTQ XL Orbitrap MS/MS	[297]
	Methyl Jasomonate	eight-plex + QSTAR, LC-TOF/TOF, LC-TripleTOF	[298]

Table S3. *Cont.*

Species	Stress	Analysis methods	Reference
Rice	Cold	2D PAGE (ProQ diamond staining for phosphoprotein) + MALDI-TOF-TOF MS	[299]
	Drought	2D PAGE + MALDI-TOF MS	[300]
	Drought	2D PAGE + LTQ-XL Orbitrap MS	[301]
	Magnaporthe oryza	2D PAGE + MALDI-TOF MS	[302]
	Magnaporthe oryza	2D PAGE + MALDI-MS/MS	[303]
	Nitrogen stress	2D PAGE + MALDI-TOF MS	[304]
	Ozone	2D PAGE + LTQ XL Orbitrap MS	[305]
	Selenium	2D PAGE + MALDI-TOF-TOF MS	[306]
	Xanothomonas campesiris pv. Oryicola	2D PAGE + MALDI-TOF-MS	[307]
Sorghum	Salt	2D PAGE + MALDI-TOF-TOF MS	[308]
Soybean	Cadmium stress	Microsomal proteins 2D PAGE + LTQ XL Orbitrap MS	[125]
	Cadmium stress	2D PAGE + LTQ XL Orbitrap MS	[309]
	Drought	2D PAGE + LTQ XL Orbitrap MS	[310]
	Flooding	2D PAGE + MALDI-TOF MS, LTQ XL Orbitrap MS	[311]
	Flooding	Proteolysis proteomics 2D PAGE + LTQ XL Orbitrap MS	[312]
	Flooding + *Bradyrhizobium japonicum*	2D PAGE + MALDI TOF MS/NanoLC MS/MS	[313]
	High temperature and humidity	2D PAGE + MALDI-TOF	[314]
	Ozone	Thio-labeling for redox proteome 2D + LC-MS/MS	[127]

Table S3. *Cont.*

Species	Stress	Analysis methods	Reference
Soybean	*Phakopora pachyrhizi*	2D PAGE + MALDI-TOF/TO	[315]
	Salt	2D PAGE + MALDI-TOF-TOF MS	[316]
	Flooding	2D PAGE + LTQ XL Orbitrap MS	[317]
	Flooding	Total protein or enriched phosphoprotein LTQ XL Orbitrap	[318]
	Flooding or low oxygen	2D PAGE + MALDI TOF MS/NanoLC MS/MS	[319]
Strawberry	Cold	2D PAGE + LC-MS/MS	[320]
	Colletorichum fragariae	2D PAGE + MALDI-TOF-TOF MS/MS	[321]
Sugarcane	Osmotic stress	2D PAGE + MALDI-TOF-MS	[322]
Tomato	*Botrytis cinerea*	2D PAGE + LC-MS/MS	[323]
Wheat	Drought	2D PAGE + MALDI-TOF MS, MALDI-TOF-TOF MS	[324]
	Drought	2D PAGE + MALDI TOF/TOF MS	[325]
	Frost	2D PAGE + MALDI-TOF MS, MALDI-TOF-TOF MS	[326]
	Fusarium graminearum	2D PAGE + LC-LTQ-Qrbitrap	[327]
	Fusarium verticillioides	SDS PAGE + LC-MS/MS	[328]
	Salt	2D PAGE + LTQ-FTICR-MS Chloroplastic proteins	[124]
	Salt	2D PAGE + MALDI TOF/TOF MS	[329]

© 2013 by the authors; licensee MDPI, Basel, Switzerland. This article is an open access article distributed under the terms and conditions of the Creative Commons Attribution license (http://creativecommons.org/licenses/by/3.0/).
